# A combined computational pipeline to detect circular RNAs in human cancer cells under hypoxic stress

**DOI:** 10.1093/jmcb/mjz094

**Published:** 2019-09-27

**Authors:** Antonella Di Liddo, Camila de Oliveira Freitas Machado, Sandra Fischer, Stefanie Ebersberger, Andreas W Heumüller, Julia E Weigand, Michaela Müller-McNicoll, Kathi Zarnack

**Affiliations:** 1 Buchmann Institute for Molecular Life Sciences, Goethe University Frankfurt, Frankfurt am Main, Germany; 2 Institute of Cell Biology and Neuroscience, Goethe University Frankfurt, Frankfurt am Main, Germany; 3 Institute for Cardiovascular Regeneration, Goethe University Frankfurt, Frankfurt am Main, Germany; 4 Department of Biology, Technical University Darmstadt, Germany; 5 Institute of Molecular Biology (IMB), Mainz, Germany

**Keywords:** circular RNA, computational pipeline, differential expression, cancer cells, hypoxia, RNA-Seq

## Abstract

Hypoxia is associated with several diseases, including cancer. Cells that are deprived of adequate oxygen supply trigger transcriptional and post-transcriptional responses, which control cellular pathways such as angiogenesis, proliferation, and metabolic adaptation. Circular RNAs (circRNAs) are a novel class of mainly non-coding RNAs, which have been implicated in multiple cancers and attract increasing attention as potential biomarkers. Here, we characterize the circRNA signatures of three different cancer cell lines from cervical (HeLa), breast (MCF-7), and lung (A549) cancer under hypoxia. In order to reliably detect circRNAs, we integrate available tools with custom approaches for quantification and statistical analysis. Using this consolidated computational pipeline, we identify ~12000 circRNAs in the three cancer cell lines. Their molecular characteristics point to an involvement of complementary RNA sequences as well as *trans*-acting factors in circRNA biogenesis, such as the RNA-binding protein HNRNPC. Notably, we detect a number of circRNAs that are more abundant than their linear counterparts. In addition, 64 circRNAs significantly change in abundance upon hypoxia, in most cases in a cell type-specific manner. In summary, we present a comparative circRNA profiling in human cancer cell lines, which promises novel insights into the biogenesis and function of circRNAs under hypoxic stress.

## Introduction

Hypoxia occurs when cells are deprived of adequate oxygen supply. Tissues react by activating endothelial cells and expressing specific growth factors to stimulate angiogenesis, thereby improving the oxygen delivery to cells. The transcriptional response to hypoxia is primarily due to the stabilization of hypoxia-inducible factors (HIFs), which control processes such as metabolic adaptation, cell survival, and apoptosis (Semenza, 2012). In addition to physiological scenarios, hypoxia is associated with several diseases, including cancer. For instance, in many solid tumors, insufficient vascularization as well as high metabolic and proliferative rates cause the formation of hypoxic cores (Semenza, 2014). The hypoxic tumor microenvironment drives cancer progression by promoting angiogenesis, malignant tumor progression, and therapy resistance (Schito and Semenza, 2016). Consequently, the expression of hypoxia markers predicts aggressive phenotypes and poor prognosis in multiple solid cancers (Graham and Unger, 2018). Extending beyond the transcriptional response, recent studies also reported large changes in alternative splicing in hypoxic cancer cells, which could modulate their oncogenic properties (Brady et al., 2017; Han et al., 2017).

Circular RNAs (circRNAs) represent a novel class of mainly non-coding RNAs, which are generated in a particular mode of alternative splicing, named back-splicing or head-to-tail splicing. In regular (linear) splicing, a 5′ splice site (or donor splice site) at the end of an exon is joined to a downstream 3′ splice site (acceptor splice site) of a subsequent exon, resulting in a linear transcript. In contrast, circRNAs are characterized by the presence of a covalent bond that links the 3′ end of an exon to an upstream 5′ end of the same or another exon. The involved splice sites (termed back-splice sites) mainly originate from genuine exons of protein-coding genes (PCGs), but may also reside within introns, untranslated regions (UTRs), and non-coding loci (Memczak et al., 2013; Guo et al., 2014; Zheng et al., 2016). Benefiting from major progress in the experimental protocols to capture circRNAs in large-scale RNA sequencing (RNA-Seq) approaches, thousands of circRNAs have been identified in various organisms from Archaea to mammals (Danan et al., 2011; Salzman et al., 2012; Wang et al., 2014). While the function of most of them remains unknown, several circRNAs have by now been described to interfere with microRNAs (miRNAs) or RNA-binding proteins (RBPs), to act as regulators of transcription or to compete with expression of their linear host transcript (Li et al., 2018). Moreover, recent studies suggested that some circRNAs can be translated to produce small peptides (Legnini et al., 2017; Pamudurti et al., 2017; Yang et al., 2017).

In humans, circRNAs are present in most tissues and cell types, and multiple transcriptome-wide studies documented cell-, tissue-, and developmental stage-specific expression patterns (Memczak et al., 2013; Salzman et al., 2013; Rybak-Wolf et al., 2015; Kristensen et al., 2017b). Intriguingly, circRNAs are often dysregulated in human cancers, including lung and breast cancer, and allow to distinguish tumors from adjacent normal tissue (Geng et al., 2018). However, little is known about the biogenesis and function of circRNAs in cancer cells under hypoxia. A recent study identified the circRNAs that are regulated in human endothelial cells upon hypoxia and revealed that circZNF292 promotes angiogenesis (Boeckel et al., 2015). The same circRNA was shown to be involved in glioma cell proliferation and tube formation (Yang et al., 2016). Similarly, the circRNA circDENND4C was found to be upregulated in human breast cancer cells (MCF-7) under hypoxia (Liang et al., 2017b). Nevertheless, the influence of hypoxia on the circRNA repertoire in cancer cells remains to be fully explored.

Here, we present a consolidated computational pipeline to detect and quantify circRNAs from RNA-Seq data. Using this approach, we profile the expression, regulation, and molecular features of circRNAs in three different human cancer cell lines under hypoxia. We predict ~12000 circRNAs, including about a quarter that were not previously described. The results show that 210 circRNAs exceed their linear counterparts in abundance and 64 circRNAs significantly change their expression upon hypoxic stress in at least one cell line. *In silico* analyses suggest an involvement of the RBP HNRNPC in circRNA biogenesis. Altogether, we identify a compendium of aberrantly expressed circRNAs in three human cancer cell lines, which promises new insights into this universal class of non-coding RNAs in the future.

## Results

### Hypoxia induces widespread changes in gene expression

In order to characterize the circRNA signature of human cancer cells and its changes in response to hypoxia, we chose three human cell lines from cervical (HeLa), breast (MCF-7), and lung (A549) cancer. To elicit hypoxic stress, MCF-7 and A549 cells were incubated for 48 h at 0.5% oxygen (O_2_), or 24 h at 0.2% O_2_ in case of HeLa cells, and compared to normoxic control cultures (21% O_2_). In order to monitor both linear and circRNAs, we sequenced total RNA depleted of ribosomal RNA (rRNA), obtaining 60–144 million reads per sample (Supplementary Table S1). As previously described, we observed extensive changes in the transcriptome, with >11000 genes that significantly altered their expression upon hypoxia (false discovery rate, FDR < 5%; Supplementary Figure S1A and B). Also, 4976 (42%) of the differentially expressed genes were shared between at least two cell lines, including classical hypoxia-induced genes, such as *CA9*, *NDRG1*, *ANGPTL4*, *VEGFA*, *PDK1*, and *BNIP3* (Chi et al., 2006; Benita et al., 2009; Lendahl et al., 2009; Sena et al., 2014). In general, genes that were upregulated showed an overrepresentation of Gene Ontology (GO) terms related to response to decreased oxygen levels, metabolic adaptation, and cell migration, while downregulated genes were enriched in terms related to ribosome biogenesis and DNA replication (*P*-value/*q*-value < 0.05; Supplementary Figure S1C). In contrast to the convergent transcriptional response, the splicing changes upon hypoxia were rather divergent, with only a few significantly regulated cassette exons overlapping between the three cell lines (30 out of 6062, 0.5%; Supplementary Figure S1D). In essence, we find that the three human cancer cell lines coincide in their gene expression profiles but differ in their alternative splicing response to hypoxic stress. Prompted by this observation, we set out to investigate the expression of circRNAs, which are generated by back-splicing as a special mode of alternative splicing.

### An improved pipeline predicts 12000 circRNAs from three human cancer cell lines

Several computational tools are available to detect circRNAs from RNA-Seq data (Szabo and Salzman, 2016; Gao and Zhao, 2018), but their predictions can vary considerably (Hansen et al., 2015; Zeng et al., 2017). We therefore decided to use a combination of two established algorithms, namely find_circ (Memczak et al., 2013) and CIRCexplorer (Zhang et al., 2014), which employ different approaches to detect reads that span the back-splice junctions (back-splice reads). In essence, find_circ uses custom scripts to scan reads that cannot be mapped continuously to the reference genome (with Bowtie2; Langmead and Salzberg, 2012) and reports separately aligning fragments in a head-to-tail arrangement. In contrast, CIRCexplorer builds on the existing splice-aware alignment algorithms TopHat-fusion (Kim and Salzberg, 2011) or STAR (Dobin et al., 2012), which predict so-called chimeric alignments, i.e. discontinuous arrangements in which the two aligned fragments are in a non-linear order.

In line with previous comparisons (Hansen et al., 2015; Zeng et al., 2017), when applied individually to a representative RNA-Seq sample, the predictions varied considerably between both tools (Supplementary Figure S2A). Even for concordantly detected circRNAs, the reported number of supporting back-splice reads was not always identical between both tools (Supplementary Figure S2B). In order to address this discrepancy, we compared the output from both tools and found that several steps can drastically influence the predictions. These included characteristics of the employed alignment algorithms (Bowtie2, TopHat2, or STAR) as well as tool-specific filters on splice-site motifs, maximum distance of back-splice sites, number of supporting back-splice reads, and exon annotation (see Table 1 for a brief comparison of features and Supplementary Note for further details). For instance, CIRCexplorer is restricted to back-splice events at splice sites that are present in the reference annotation, whereas find_circ allows the *de novo *prediction of cryptic splice sites anywhere within transcripts, including introns. Conversely, find_circ limits the accepted back-splice events to canonical GU/AG pairs, which represent the most common motifs at donor/acceptor splice sites (Burset et al., 2000; Abril et al., 2005).

**Table 1:** Main features of tools adopted in this study to detect circRNAs.

	**find_circ**	**CIRCexplorer**	**Our pipeline**
**Alignment algorithm**	Bowtie2	STAR	Both
**Splice site motifs**	GU/AG	Any	GU/AG + GC/AG
**Splice sites**	Annotated + *de novo*	Annotated	Annotated + *de novo*
**Genomic distance of back-splice sites**	≤100 kb	Within single gene	≤100 kb
**Expression filter**	2 distinct reads (sequence-based)	Any 2 reads	2 distinct reads (coordinate-based)

**Figure 1:**
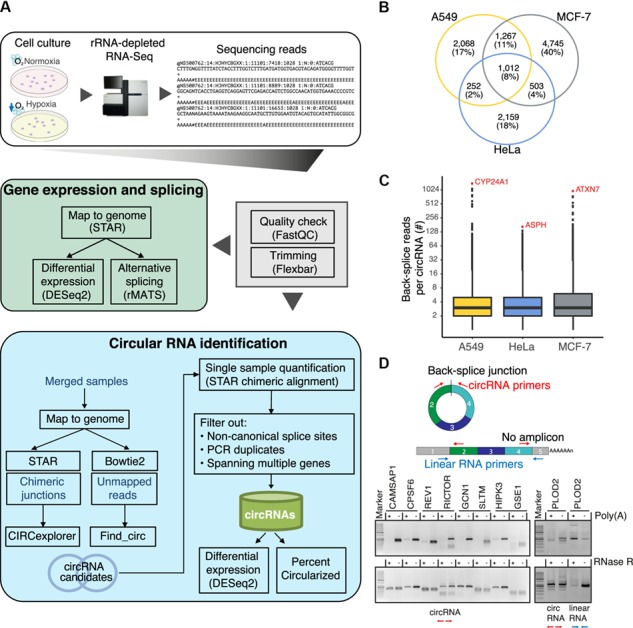
Identification and validation of circRNAs. (**A**) Consolidated pipeline for circRNA identification and expression analysis from rRNA-depleted RNA-Seq data of normoxic and hypoxic cancer cells. The initial circRNA predictions by find_circ (Memczak et al., 2013) and CIRCexplorer (Zhang et al., 2014) are combined and rigorously filtered to obtain a comprehensive catalog of circRNAs. In parallel, linearly mapped reads are used for the analysis of differential gene expression and alternative splicing. (**B** and **C**) Analyses of the complete catalog of 12006 circRNAs (≥2 supporting back-splice reads in any sample). (**B**) Venn diagram showing the overlap of circRNAs identified in A549, HeLa, and MCF-7 cells. (**C**) Boxplot showing the number of back-splice reads from each cell line. circRNAs hosted by the genes *CYP24A1* (circBase ID hsa_circ_0060927), *ASPH* (hsa_circ_0084615), and *ATXN7* (hsa_circ_0007761) were the top expressed circRNAs in A549, HeLa, and MCF-7 cells, respectively. (**D**) Validation of circularity for 9 circRNAs in HeLa cells. Due to their lack of a poly(A) tail and free ends, circRNAs are only amplified from the polyA(−) fraction and resistant to the exonuclease cleavage (RNase R). Top: schematic of oligonucleotides used in RT-PCR to amplify the circRNA (red) or the related linear transcript isoform (blue). Bottom: RT-PCR products for 9 circRNAs using divergent oligonucleotides after polyA(+) selection or RNase R treatment. Oligonucleotides amplifying the linear *PLOD2* transcript were used as control.

Based on these conceptual differences, we designed a pipeline that combines the best features of both tools to obtain a comprehensive catalog of circRNAs (Figure 1A; Supplementary Figure S2; see Supplementary Note for more details). Briefly, for each cell type, the sequencing reads were merged and mapped to the human genome (version GRCh38/hg38) with Bowtie2 (Langmead and Salzberg, 2012) and STAR (Dobin et al., 2012). Unmapped reads from Bowtie2 served to identify circRNAs with find_circ as described in (Memczak et al., 2013). Chimeric alignments from STAR were used to detect circRNAs with CIRCexplorer. We combined the initial circRNA predictions by both tools and then filtered out inconsistencies and detection artifacts of either algorithm. Finally, we harmonized the quantification estimates by recounting back-splice reads for all circRNAs from the STAR chimeric alignments. Putative PCR duplicates were flagged based on genomic mapping positions rather than read sequences. As a cutoff for a circRNA to be present in a given cell line, we demanded a minimum of two distinct back-splice reads in at least one replicate. For the subsequent circRNA abundance estimates, all back-splice reads were taken into account. We evaluated the performance of our approach by estimating the number of false-positives in a published dataset in which circRNAs were specifically enriched by RNase R digestion (Jeck et al., 2013; see Supplementary Note for more details). We found that the precision of our approach was higher than for find_circ and comparable to CIRCexplorer (Supplementary Figure S2F). Overall, we conclude that our pipeline provides reliable predictions of circRNAs.

**Figure 2:**
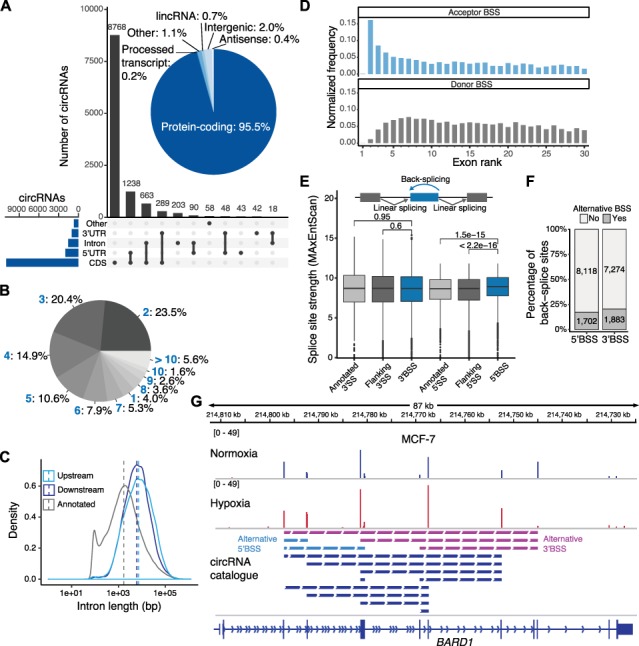
Genomic features of circRNAs. (**A**) circRNAs are mainly produced from CDS of PCGs. Bar chart displaying the frequency of transcript regions harboring the 5′ and 3′ back-splice sites that come together in a given circRNA. Inlay: pie chart showing the genomic origin of circRNAs. (**B**) Most circRNAs comprise < 5 putative internal exons. Pie chart showing the percentage of circRNAs with a given number of annotated exons (blue) located between the back-splice sites. (**C**) Back-splice sites are flanked by longer introns than average. Density plot showing distribution of intron lengths for circRNA-flanking introns (upstream and downstream) compared to all introns annotated in GENCODE version 24. Dashed lines indicate median. (**D**) Distribution of exon ranks involved in circRNA formation as acceptor (top) or donor splice site (bottom). The normalized frequency was obtained by dividing the frequency of a certain exon rank as acceptor/donor splice site by the number of genes containing at least this number of exons +1 (GENCODE version 24). Only circRNAs produced from PCGs and with both back-splice sites within the same annotated transcript were evaluated (*n* = 9676). (**E**) Strength of 5′ back-splice sites is significantly higher than at flanking 5′ linear splice sites and 2000 randomly selected 5′ linear splice sites (GENCODE version 24). Splice site strength estimated with MaxEntScan (Yeo and Burge, 2004). Same circRNA selection as in **D**, further excluding circRNAs involving first/last exons of annotated transcripts, resulting in 9664 circRNAs. (**F**) More than 20% of back-splice sites participate in alternative back-splice events. (**G**) Alternative 3′ (magenta) and 5′ (light blue) back-splicing generates 14 distinct circRNA isoforms from the *BARD1* gene in A549, HeLa, and MCF-7 cells. Genome browser view of *BARD1* gene, showing chimeric alignments (back-splice reads) from RNA-Seq of MCF-7 cells under normoxic and hypoxic conditions. Dashed lines below connect the paired back-splice sites. The *BARD1* gene is located on the minus strand.

Applying our pipeline to the RNA-Seq datasets from the three human cancer cell lines, we identified a total of 12006 circRNAs (Figure 1B; Supplementary Table S2). Despite a similar sequencing depth, the number of detected circRNAs was considerably higher in MCF-7 cells (7527 circRNAs) compared to A549 cells (4599; Supplementary Table S1). Accordingly, circRNAs in MCF-7 were supported by more back-splice reads compared to A549 cells (Supplementary Figure S3A). This may reflect not only physiological differences in circRNA abundance but also experimental variation, e.g. in rRNA depletion efficiency during library preparation. In HeLa cells, the sequencing depth was generally lower, resulting in fewer detected circRNAs (3926) that were supported by less back-splice reads. As previously observed (Memczak et al., 2013; Salzman et al., 2013; Guo et al., 2014; Zhang et al., 2014), the majority of circRNAs in all cell lines were lowly abundant, reflected in <5 back-splice reads (Figure 1C). Nevertheless, we detected many abundant circRNAs (1392 circRNAs with ≥10 back-splice reads in at least one replicate). The most highly expressed circRNAs originated from the genes *ASPH, ATXN7*, and *CYP24A1* in HeLa, MCF-7, and A549 cells, respectively, each represented by >150 back-splice reads in a single replicate (Figure 1C).

In order to test our predictions, we performed a series of experimental validations. First, we confirmed the presence and circularity of 10 circRNAs in HeLa cells using reverse transcription PCR (RT-PCR) with divergent primer pairs flanking the back-splice junctions (Figure 1D; Supplementary Figure S2G). In addition to the presence of amplification products, we confirmed that all tested circRNAs lacked a poly(A) tail and were resistant to RNase R treatment, further supporting their circularity (Figure 1D; Supplementary Figure S2G).

Comparison to the circRNA databases circBase (Glažar et al., 2014) or circRNADb (Chen et al., 2016) revealed that 2844 of the detected circRNAs (24%) had not been reported previously. For instance, we predicted novel circRNAs from the genes *PICALM*, *SPIDR*, and *HUWE1*, which were present in all cell lines and often supported by >20 back-splice reads (Supplementary Table S2). In summary, our combined pipeline yielded a comprehensive catalog of 12006 circRNA candidates in three human cancer cell lines under normoxic and hypoxic conditions combined.

### circRNAs predominantly arise from internal exons of PCGs

In order to characterize the circRNAs in more detail, we first investigated their genomic origin. In line with previous observations, ~95% of the 12006 circRNAs originated from PCGs, with 91% of all back-splice sites residing in the coding sequence (CDS; Figure 2A). This meant that 21% (4252) of all annotated PCGs (19940 genes; GENCODE version 24) hosted at least one circRNA in any of the analyzed cell lines. The circRNA-producing PCGs were significantly longer than average PCGs (*P*-value < 2.2E−16, Wilcoxon rank sum test; Supplementary Figure S3B), and included a higher number of exons (average of 21 exons compared to 12, respectively). The number of annotated exons between the back-splice sites ranged between 1 and 40 exons, with a median of 4 exons that were potentially included into a single circRNA (Figure 2B; Lei et al., 2018). For such multi-exon circRNAs (*n* = 9201), the median distance of back-splice sites in the unspliced pre-mRNA was almost 10 kb (Supplementary Figure S3C; Lei et al., 2018). While the introns flanking the back-splice sites were generally longer than average annotated introns (Figure 2C; Zhang et al., 2014), the back-spliced exons were similar in length compared to all exons from PCGs (median 125–127 bp vs. 143 bp for all annotated exons; Supplementary Figure S3D). Notable exceptions were single-exon circRNAs (*n* = 475), which showed a median exon length of 390 bp (Supplementary Figure S3C; Lei et al., 2018).

Along the pre-mRNAs, back-splicing preferentially occurred at the first genuine acceptor splice site in the nascent transcript, i.e. the 3′ splice site of the second exon, irrespective of its splice site strength (MaxEnt score; Figure 2D and E; Yeo and Burge, 2004). In contrast, no exon position was preferred for the donor back-splice sites (Figure 2D). However, we found that 5′ splice site strength at donor back-splice sites was significantly higher than at flanking or randomly selected 5′ splice sites that did not engage in back-splicing (Figure 2E). This observation implied that spliceosome assembly at the 5′ splice site might influence the decision of circular vs. linear splicing.

Even from a conservative perspective based on back-splice junctions, more than half of the host genes showed alternative back-splicing, i.e. they produced multiple circRNA isoforms (Supplementary Figure S3E; Zhang et al., 2016). For instance, we detected four alternative isoforms of circZNF292, including the previously published intronic circZNF292 (hsa_circ_0004383) from a back-splice site in the cryptic exon 1a (Boeckel et al., 2015), as well as additional variants taking both back-splice sites from genuine exons of *ZNF292*. Alternative back-splicing ranged from 2 up to 36 distinct circRNAs for the *TRIM37* gene (Supplementary Table S2). In many cases, we still observed a strong prevalence of one single isoform over the others (Supplementary Figure S3F). In general, alternative back-splicing choices occurred with almost identical frequency at donor and acceptor splice sites (Figure 2F), as exemplified by the *BARD1* gene which generated up to 14 different circRNA isoforms (Figure 2G). In addition to alternative back-splicing, the circRNA repertoire is likely further expanded by internal alternative splicing events within the circRNAs, which were not taken into account in this analysis.

### The circRNA signatures differ between cell lines

**Figure 3:**
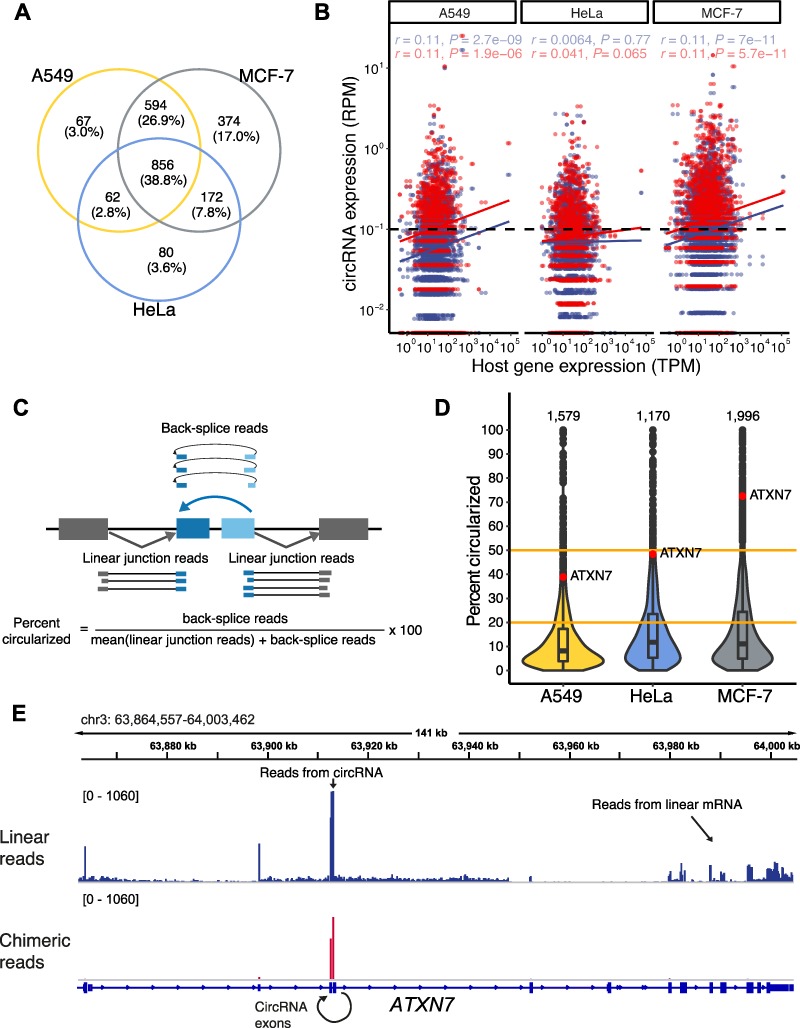
CircRNA profiles in human cancer cells. (**A**) Comparison of high-confidence circRNAs across A549, HeLa, and MCF-7 (supported by a minimum of 5 back-splice reads in any 2 samples). Most circRNAs are expressed in at least two cell types. A similar partition occurs when a further filter on minimum expression of the host gene is applied (Supplementary Figure S3H). (**B**) Scatter plot comparing the expression of circRNAs in back-splice RPM to the expression of the host gene in TPM. circRNA expression does not generally reflect the abundance of the host gene. Mean expression across replicates is shown for each cell line under hypoxic (blue) and normoxic (red) conditions. Linear regression lines and Pearson correlation coefficients with associated *P*-values are reported. (**C**) Scheme showing how ‘percent circularized’ metric is computed from back-splice reads and reads spanning the corresponding linear splice junctions. (**D**) In total, 210 circRNAs are more abundant than their linear counterparts, as exemplified by circATXN7 (hsa_circ_0007761; labeled in orange; Huang et al., 2019) in MCF-7 cells. Violin plot shows distribution of ‘percent circularized’ values for circRNAs from the three cell lines (mean per cell line across all replicates and conditions). Orange lines indicate circRNAs with >20% and 50% relative abundance. (**E**) Genome browser view of *ATXN7* gene showing RNA-Seq data from MCF-7 cells under normoxic conditions. Coverage of chimeric alignments (back-splice reads, red, bottom) documents back-splicing of exon 4 to exon 3 to produce circATXN7. The high abundance of circATXN7 is reflected in a peak in the coverage of linearly mapped reads (blue, top) corresponding to internal regions of the circRNA, while the remainder of the linear transcript shows less coverage.

Out of the total of 12006 circRNAs, only 25% were shared among all three cell lines, suggesting that most circRNAs (75%) were expressed in a cell type-specific manner (Figure 1C). However, as outlined above, the majority of circRNAs were supported by a small number of reads, making it difficult to compare their expression. We therefore defined a ‘high-confidence set’ of 2205 circRNAs, for which we required a minimum of five supporting back-splice reads in any two samples. As a consequence, the fraction of cell line-specific circRNAs dropped to 24% (Figure 3A). From these circRNAs, a substantial part seemed to originate from genuine cell line-specific back-splicing events rather than differentially expressed host genes, since an additional filter on host gene expression did not change their relative contribution (transcripts per million, TPM ≥ 5 in any sample of a given cell line; Supplementary Figure S3G). The remaining 76% of circRNAs were detected in at least two out of three cell lines (Figure 3A). GO analysis of 690 genes hosting the circRNAs shared among the three cell lines documented an overrepresentation of terms ‘covalent chromatin modification’, ‘establishment or maintenance of cell polarity’, and ‘microtubule skeleton association’ (Supplementary Figure S3H), indicating that they represent highly expressed genes with potential housekeeping functions.

**Figure 4:**
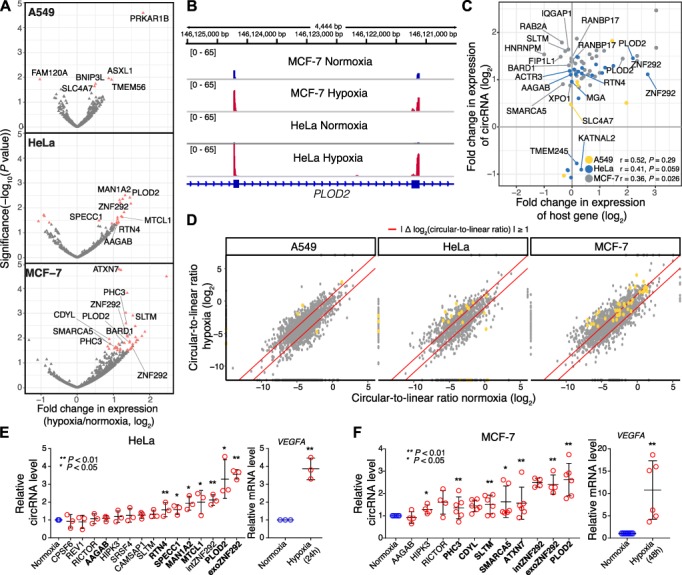
Hypoxia-induced changes in circRNA levels. (**A**) Sixty-four circRNAs significantly change in abundance upon hypoxia. Volcano plots show log_2_-transformed moderated fold changes in expression (hypoxia over normoxia, taken from DESeq2) of circRNAs in the three cancer cell lines against associated *P*-values (−log_10_). Differentially expressed circRNAs are highlighted in red (FDR < 0.05). Labels indicate circRNAs that were validated by RT-qPCR in **E** and **F**. Only high-confidence circRNAs with at least 5 reads in any 2 samples of a single cell line were tested for differential expression. (**B**) circPLOD2 is consistently upregulated upon hypoxia. Genome browser view of exons 2–3 of the *PLOD2* gene. The gene is located on the minus strand. Chimeric alignments (back-splice reads) from RNA-Seq data for MCF-7 and HeLa cells under normoxic and hypoxic conditions are shown. (**C**) Many upregulated circRNAs originate from upregulated host genes. Scatterplot compares log_2_-transformed moderated fold changes in expression (hypoxia over normoxia, taken from DESeq2) of 64 hypoxia-regulated circRNAs and their host genes in the three cancer cell lines (indicated by color). Pearson correlation coefficients and associated *P*-values are given for each cell line. Selected circRNAs are labeled. (**D**) Most circRNAs do not change in back-splicing rate between conditions. Scatterplots compare circular-to-linear ratios (CLRs) of all high-confidence circRNAs in the three cell lines under hypoxic and normoxic conditions. Red lines indicate >2-fold change in CLR between conditions. The hypoxia-regulated circRNAs (significantly changed in overall abundance according to DESeq2) in each cell line (as shown in **A**) are highlighted in orange. (**E** and **F**) Expression changes of hypoxia-regulated (bold) and control (regular) circRNAs in HeLa (**E**) and MCF-7 (**F**) cells kept in hypoxic conditions (24 h and 48 h, respectively). Dot plot shows relative circRNA levels (over normoxia) based on RT-qPCR normalized to U6 snRNA for HeLa and *P0*(*MPZ*) for MCF-7 (Supplementary Figure S4F). Mean and standard deviation of the mean are shown together with red circles for replicate measurements. In HeLa cells, all seven expected circRNAs were significantly upregulated (*n* = 3, *P < 0.05, **P < 0.01). We additionally included intronic circZNF292, known to be hypoxia-induced in HUVEC cells (Boeckel et al., 2015), although its regulation did not reach significance in our RNA-Seq data analysis. In MCF-7 cells, six of the seven expected circRNAs were significantly upregulated (*n* ≥ 3, *P < 0.05, **P < 0.01), as well as circHIPK3 that was not found as significantly regulated in the RNA-Seq data. Upregulation of *VEGFA* mRNA served as control for hypoxia treatment.

Beyond this general association, we observed only a weak quantitative correlation between the expression of circRNAs and their respective host genes (Figure 3B). This suggested that in many cases, circRNA abundance did not just reflect host gene expression, but was influenced by independent parameters, such as varying degrees of back-splicing or circRNA stability. To directly assess back-splicing efficiency, we used the ‘percent circularized’ metric based on the number of linear and back-splice junctions in the RNA-Seq reads to estimate the relative abundance of circRNAs in comparison to all isoforms including the same exon (Figure 3C). As expected, most circRNAs represented minor transcript isoforms of their host genes. Nevertheless, 210 circRNAs were more abundant than their linear counterparts in at least one cell line (Figure 3D). For example, a circRNA comprising exons 3 and 4 was the most abundant isoform from the *ATXN7* gene in MCF-7 cells (Figure 3D and E). We therefore concluded that different regulatory processes can direct the expression of circRNAs and linear transcript isoforms and that back-splicing strongly varies in efficiency between host genes.

### Several circRNAs change in abundance upon hypoxia

Next, we addressed whether the hypoxic stress modulates circRNA abundance in the cancer cell lines. First, we did not observe a difference in the overall number of circRNAs present under hypoxic and normoxic conditions (Supplementary Table S1). In order to test for individual circRNAs that significantly change in abundance, we performed a combined DESeq2 analysis (Love et al., 2014) of linear and circRNAs to improve library size estimation, normalization, and statistical power. We identified a total of 64 circRNAs that were significantly regulated upon hypoxia in any of the analyzed datasets, ranging between 6 and 38 per cell line (FDR < 10%; Figure 4A; Supplementary Figure S4A and Table S3). Out of the 64 circRNAs, only six were detected as downregulated within the timeframe of the experiment, probably due to the high intrinsic stability of circRNAs. Moreover, 97% were regulated in a cell type-specific manner (62 out of 64 circRNAs). The exceptions were circPLOD2 (Figures 1D and 4B) and the alternative isoform of circZNF292 (circZNF292_exonic; Supplementary Figures S2G and S4B), which significantly increased in abundance in both HeLa and MCF-7 cells. circZNF292 was previously reported to be upregulated upon hypoxia in endothelial cells (Boeckel et al., 2015), underlining its robust response in different physiological contexts. We did not recover a significant regulation of circDENND4C as it had been reported previously (Liang et al., 2017b) due to the low overall counts for this circRNA.

In order to control whether the changes in circRNA abundance reflected host gene regulation, we compared circRNA and host gene expression for the hypoxia-induced circRNAs. Although not quantitatively correlated, many of the hypoxia-induced circRNAs still originated from upregulated genes, indicating that the observed circRNA upregulation could be related to an increased transcription of the host gene (Figure 4C). For other upregulated circRNAs, such as circBARD1 and circRANBP17, the level of the host gene remained stable. Other than recently suggested, we did not observe evidence that circRNAs were produced from read-through transcription of upstream genes (Liang et al., 2017a; Supplementary Figure S4C).

In order to directly assess changes in back-splicing, we compared normoxic and hypoxic cells at the level of ‘circular-to-linear ratio’ (CLR), i.e. the number of reads containing back-splice vs. linear exon–exon junctions involving the same splice site. We found that although back-splicing rates varied considerably between circRNAs, they were stable between replicates and cell lines (Supplementary Figure S4D and E), indicating that the back-splicing efficiency was determined by the molecular features at each locus. The majority of the 64 circRNAs that changed their overall abundance under hypoxia displayed little changes at the level of CLR (Figure 4D), further supporting the notion that the observed upregulation was coupled to transcriptional activation of the host gene rather than differential back-splicing. Despite this general trend, circRNAs like circHNRNPM and circRAPGEF5 showed a >2-fold increase in back-splicing in MCF-7 cells (Supplementary Table S3).

In order to independently support these results, we used quantitative PCR (RT-qPCR) to measure the expression of two circZNF292 isoforms and 13 further circRNAs, adding up to eight stable and seven significantly changed circRNAs according to our RNA-Seq analyses. Experiments were performed in HeLa and/or MCF-7 cells. RT-qPCR with divergent primers spanning the back-splice junctions confirmed a significant change upon hypoxia for all seven regulated circRNAs in HeLa cells and 6 out of 7 circRNAs in MCF-7 (plus circHIPK3 which was significantly changing in RT-qPCR but stable in RNA-Seq; Figure 4E and F). In line with the RNA-Seq results (Figure 4A; Supplementary Figure S4A), circPLOD2 and both circZNF292 isoforms displayed a strong and consistent upregulation in both tested cancer cell lines. Altogether, we identified several circRNAs that significantly responded to hypoxic stress in the cancer cell lines.

### circRNAs can be generated via intron complementarity

**Figure 5:**
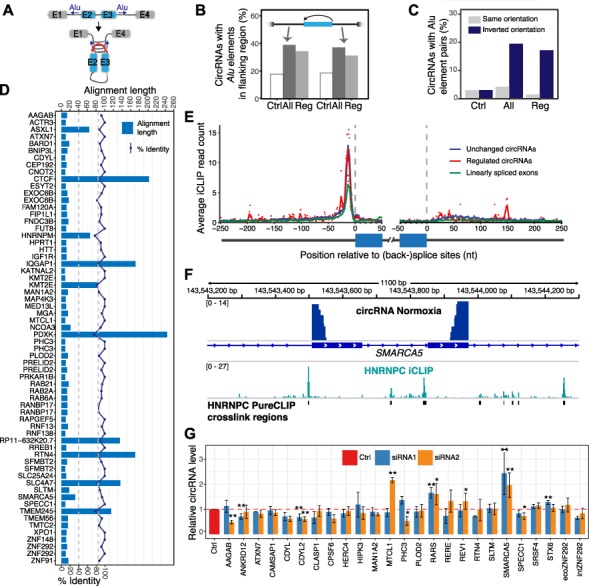
Biogenesis of hypoxia-regulated circRNAs. (**A**) Schematic visualization of how pairing of inverted *Alu* elements in flanking introns can promote circRNA formation. (**B**) circRNA-flanking introns are enriched in *Alu* elements. Barchart shows the percentage of introns with *Alu* elements in a 500-bp window next to splice sites of annotated internal exons (Ctrl, white) compared to back-splice sites of all (All, dark gray) and hypoxia-regulated (Reg, light gray) circRNAs. (**C**) circRNA-flanking *Alu* element pairs are more often in inverted orientation. Barchart shows the percentage of *Alu* element pairs in the same (gray) or inverted (blue) orientation. Exon categories and windows as in **B**. (**D**) Some regulated circRNAs harbor extended complementary sequences in their flanking introns. Barchart depicts that length and identity of longest local alignment (left and right scales, respectively) from pairwise alignments of flanking introns (500-bp window) for the 64 hypoxia-regulated circRNAs. The upper and lower dashed lines indicate 85% identity and a minimum alignment length of 40 nt, respectively. Mutation experiments demonstrated that inverted repeats of 30–40 nt are sufficient to promote circularization (Liang and Wilusz, 2014). (**E**) HNRNPC binding shows more binding at back-splice sites compared to linearly spliced exons. Metaprofile of HNRNPC binding (iCLIP) in a 300-nt window around back-splice sites, including 250 nt intron and 50 nt into the circularized exons. circRNAs of the high-confidence set expressed in HeLa (*n* = 1133) were separated into hypoxia-regulated and non-regulated circRNAs and compared to 4853 linear exons from expressed PCGs that do not undergo circularization. For each position, the mean coverage in each set is shown by a dot. Lines were smoothed with locally weighted polynomial regression (loess, span = 0.05). (**F**) HNRNPC binds upstream of the 3′ back-splice site of circSMARCA5, which is upregulated upon *HNRNPC* depletion. Genome browser view shows section of the *SMARCA5* gene including RNA-Seq data (chimeric alignments) for HeLa cells under normoxic conditions (top) and HNRNPC iCLIP data from HeLa cells. Binding sites predicted by PureCLIP are shown below. (**G**) Expression changes of a panel of 25 circRNAs upon *HNRNPC* depletion with two independent siRNAs in HeLa cells. Barchart shows the relative circRNA levels based on RT-qPCR normalized to U6 snRNA. Data were shown as mean ± SD. circCDYL2, circRARS, and circSMARCA5 were significantly deregulated (*n* = 3, *P < 0.05, **P < 0.01).

As a first step toward the molecular mechanisms underlying circRNA biogenesis and regulation, we revisited the molecular features of circRNAs in our samples. It was shown that back-splicing can be initiated by complementary sequences in the flanking introns that enclose the circularizing exons and potentially bring the back-splice sites in close proximity (Figure 5A). Such complementary sequences commonly originate from repetitive elements, in particular *Alu* element retrotransposons (Zhang et al., 2014). In line with a contribution to circRNA biogenesis, the introns flanking the back-splice sites in our dataset were longer than average annotated introns and displayed an elevated frequency of *Alu* elements (RepeatMasker annotation; Figures 2C and 5B). Moreover, *Alu* element pairs flanking the back-splice sites preferentially occurred in inverted orientation, thereby enabling complementary base pairing (Figure 5C). Focusing on the 64 hypoxia-regulated circRNAs, we complemented these analyses with an unbiased approach based on pairwise local alignments of the flanking introns. We detected complementary regions in seven circRNA-flanking intron pairs, which all originated from *Alu* element insertions (Figure 5D). Inverted *Alu* elements thus occurred with similar frequency in hypoxia-regulated and other circRNAs in our dataset (*Chi*-squared test, *P*-value > 0.1; Figure 5B and C), supporting the general notion that complementary *Alu* elements may drive circRNA biogenesis in some, but not all cases.

Lariat formation from exon skipping has been proposed as another way to produce circRNAs from a pre-mRNA (Kelly et al., 2015; Khan et al., 2016). However, we could not detect linear transcripts skipping the circularized exons for any of the hypoxia-regulated circRNA, indicating that this mechanism did not play a prominent role in this scenario. Alternatively, the skipped transcripts could be unstable and quickly degraded.

### The RBP HNRNPC can regulate circRNA biogenesis

It has been reported that RBPs can influence circRNA formation (Ashwal-Fluss et al., 2014; Conn et al., 2015; Errichelli et al., 2017). In order to investigate this, we first predicted putative RBP binding sites in the introns flanking the back-splice sites of the 2205 circRNAs in the high-confidence set using Find Individual Motif Occurrences (FIMO) (Grant et al., 2011). Among the most frequently represented RBPs at hypoxia-regulated circRNAs were HuR, HNRNPC, and PABPC4 (Supplementary Figure S5A and B). Of these, HuR had already been linked to circRNA function, although not at the level of biogenesis (Abdelmohsen et al., 2017). Neither of the three RBPs showed a preference for the hypoxia-regulated compared to other circRNAs, suggesting a more general function in circRNA biogenesis.

Since we had previously found HNRNPC to directly interfere with 3′ splice site recognition (König et al., 2010; Zarnack et al., 2013), we decided to investigate its role in more detail. Using published iCLIP datasets from HeLa cells (Zarnack et al., 2013), we assessed the binding of HNRNPC around back-splice sites and control splice sites from linearly spliced exons. HNRNPC showed a predominant peak immediately upstream of the 3′ back-splice sites, as exemplified for circSMARCA5 (Figure 5E and F). In line with the in silico predictions, HNRNPC was equally enriched on hypoxia-regulated and other circRNAs. Notably, the peak in *HNRNPC* binding was substantially increased at back-splice sites compared to linearly spliced exons, suggesting a role of HNRNPC in circRNA biogenesis. In order to test this hypothesis, we used two independent siRNAs to deplete *HNRNPC* from HeLa cells and confirmed the functional knockdown (KD) by measuring the splicing changes in four previously described HNRNPC target exons (Zarnack et al., 2013; Supplementary Figure S5C–E). Next, we tested a panel of 25 circRNAs, for which we had primers available. Using this untargeted approach, we identified three circRNAs significantly changed in both *HNRNPC* KDs, including the downregulation of circCDYL2 as well as the upregulation of circRARS and circSMARCA5 (Figure 5G). Although we cannot rule out confounding effects from changes in the underlying transcript abundance, it is tempting to speculate that HNRNPC acts as a regulator of circRNA biogenesis, possibly via direct binding at the 3′ back-splice sites (see Discussion).

### The majority of circRNAs do not seem to act as miRNA sponges

It has been shown that some circRNAs present numerous miRNA target sites and thereby act as miRNA sponges to indirectly regulate gene expression (Hansen et al., 2013; Memczak et al., 2013; Wang et al., 2016; Zheng et al., 2016). To test whether circRNAs in the analyzed cancer cells might exert such a function, we predicted miRNA target sites within the putative internal circRNA sequences using the miRanda algorithm (Enright et al., 2003). We found that in general, circRNAs did not show a higher density of miRNA target sites compared to randomly selected annotated 3′ UTRs and CDS regions (Supplementary Figure S5F). The same held true for the 64 hypoxia-regulated circRNAs, for which the number of predicted miRNA target sites directly followed the putative length of the fully spliced circRNA (Supplementary Figure S5G). This was different for the control circRNA CDR1as/ciRS-7, which was previously shown to harbor dozens of functionally relevant target sites for miR-7 and miR-671 (Hansen et al., 2013; Memczak et al., 2013). In line with other studies (Guo et al., 2014; Boeckel et al., 2015; You et al., 2015), these analyses suggest that miRNA sequestration is not a predominant mechanism of action of circRNAs in the analyzed human cancer cell lines.

## Discussion

circRNAs are a special class of non-coding RNAs, which receive increasing recognition for their unique RNA biology, their molecular and physiological functions, and their distinctive expression patterns. Due to their underrepresentation in classical transcriptome profiling protocols based on polyA(+) RNA-Seq, their abundance had long been overlooked. Moreover, since circRNAs overlap in most parts with their linear RNA counterparts, they offer little discriminative sequence information for reliable detection, and the *de novo* prediction of back-splicing events at cryptic splice sites remains particularly challenging (Szabo and Salzman, 2016). In recent years, numerous algorithms have been developed to predict and quantify circRNAs from total RNA-Seq data, each offering unique advantages, but also high levels of false positives (Szabo and Salzman, 2016). A combination of tools is therefore advised to gain highest accuracy in circRNA predictions (Hansen, 2018).

Here, we present a combined computational pipeline based on the widely used algorithms find_circ and CIRCexplorer (Memczak et al., 2013; Zhang et al., 2014). The two tools complement each other, as they rely on different alignment algorithms (Bowtie2 and STAR) and conceptual approaches. While the custom approach by find_circ tends to suffer from false positives due to inaccurate back-splice junction assignments, CIRCexplorer is limited to exon coordinates from reference annotation. In order to gain most in terms of sensitivity and specificity, we combine the output from both tools and then rigorously filter to counteract the tool-specific weaknesses. Finally, we recount the supporting back-splice reads for all predicted circRNAs in the chimeric alignments from STAR to obtain consistent quantitative estimates. Our pipeline thus outputs a comprehensive list of accurately quantified circRNAs as an entry point into downstream analyses.

Using our pipeline, we identified ~12000 circRNAs in three human cell lines from cervical, lung, and breast cancer patients. About one quarter had not been reported before. In our study, many circRNAs were selectively expressed in just one cell line, suggesting that the analyzed cancer cells displayed a unique circRNA signature (Xia et al., 2016). In line with this notion, a recent study reported >1000 circRNAs that were deregulated in tumors, but not neighboring tissue, from 51 breast cancer patients (Lü et al., 2017). Even if some of these circRNAs may represent splicing by-products and reflect general differences in gene expression, their abundant appearance could provide meaningful signatures to indicate cancer incidences.

Beyond their abundant expression in cancer cells, an increasing number of circRNAs were reported to promote oncogenic mechanisms. For instance, circGFRA1 was previously found to be highly expressed in triple-negative breast cancer patients where it correlated with poor clinical outcome (He et al., 2017). In our study, this circRNA was highly abundant in MCF-7 cells, but absent from the other two cancer cell lines, although this largely reflected the expression of the host gene (hsa_circ_0005239; Supplementary Table S2). An association with tumor progression and malignancy was also reported for circHIPK3 and circPIP5K1A (Geng et al., 2018), which were both present in at least one cancer cell line in our analysis.

Because of their high abundance and their inherent stability due to their covalently closed structure, circRNAs offer promising targets for cancer diagnosis, prognosis, and therapy (Lü et al., 2017; Kristensen et al., 2017a). In particular, with advanced tumor progression, circRNAs may serve as robust indicators of a hypoxic tumor microenvironment. Moreover, several circRNAs have been implicated in oncological pathways, where their appearance correlates with poorer prognosis (Verduci et al., 2019). In addition, circRNAs have been explored for protein expression (Wesselhoeft et al., 2018) and miRNA sequestration (Jost et al., 2018), which may open new therapeutic avenues in the future.

Using our analysis pipeline, we identified 64 circRNAs that are significantly deregulated upon hypoxia in the studied cancer cell lines (Supplementary Table S3). In particular, we observed a consistent and robust upregulation of circZNF292 isoforms. The intronic circZNF292 variant (hsa_circ_0004383) was among the first circRNAs found as regulated upon hypoxia (Boeckel et al., 2015) and associated with cell proliferation and tube formation in human glioblastoma (Yang et al., 2016). An aberrant expression in glioblastoma was also detected for the second shared hypoxia-regulated circRNA circPLOD2 from our analysis (hsa_circ_0122319). Moreover, our analyses revealed a 3–5-fold upregulation of two circPRELID2 isoforms in hypoxic MCF-7 cells. In a previous study, one of these circRNAs (hsa_circ_0006528) was highly expressed in chemotherapy-resistant MCF-7 cells, indicating a potential use as therapeutic target or prognostic biomarker for therapy response (Gao et al., 2017). The fact that only 6 circRNAs were found as downregulated in our study is likely attributable to the increased stability of circRNAs compared to linear RNAs, which can mask changes when looking at short-term stimuli. In addition, specific mechanisms of circRNA degradation have been described, such as the slicing of CDR1as/ciRS-7 by miR-671 as part of intricate regulatory feedback loops (Hansen et al., 2011; Kleaveland et al., 2018).

Multiple RBPs, such as MBNL, QKI, FUS, and SR proteins, can impact on circRNA formation (Ashwal-Fluss et al., 2014; Conn et al., 2015; Kramer et al., 2015; Errichelli et al., 2017). Our data suggest HNRNPC as a novel RBP involved in circRNA biogenesis. Using an untargeted approach, we identified three circRNAs that responded to *HNRNPC* depletion in HeLa cells. Although we cannot rule out changes in transcript abundance, several mechanisms are conceivable how HNRNPC may exert this regulation at the level of back-splicing. For instance, HNRNPC was previously shown to interfere with U2AF2 binding at genuine and cryptic 3′ splice sites to prevent exon inclusion, thereby maintaining splicing fidelity (Zarnack et al., 2013). With respect to the HNRNPC-repressed circRNAs, it is tempting to speculate that similar mechanisms are in place to interfere with back-splicing. In addition, HNRNPC may also exert a regulatory function via *Alu* elements, which often act as drivers of RNA circularization (Chen, 2016). Notably, HNRNPC was previously shown to extensively bind to *Alu* elements in nascent transcripts (Zarnack et al., 2013) and could thereby suppress the pairing of inverted *Alu* repeats. In line with this notion, siRNA-mediated *HNRNPC* depletion in MCF-7 cells was recently reported to trigger an increased abundance of double-stranded RNA regions, which were highly enriched in *Alu* elements (Wu et al., 2018). It remains to be investigated whether, as in linear alternative splicing, the positioning of HNRNPC relative to the back-splice sites as well as its integration with further regulatory elements may determine the regulatory outcome for each circRNA.

In summary, we performed a comparative circRNA profiling of three human cancer cell lines under hypoxic stress conditions. A detailed knowledge about the expression and putative functions of circRNAs in human physiology and disease will open possibilities for the development of new disease biomarkers and therapeutic approaches in the future.

## Materials and methods

### Cell culture and treatments

HeLa cells were cultured in 10-cm plates in high glucose (4.5 g/L) DMEM (Sigma Aldrich) supplemented with 10% FBS, 100 U/ml penicillin, and 100 μg/ml streptomycin (Pen Strep, all from Thermo Fisher Scientific). The cells were plated and grown in a normal incubator until they reached 60% confluency (21% O_2_, 37°C, 5% CO_2_), and then either kept in the normal incubator (normoxic conditions) or transferred to a hypoxia chamber (0.2% O_2_, 37°C, 5% CO_2_) for 24 h.

A549 (DSMZ no. ACC-107) and MCF-7 (DSMZ no. ACC-115) cells were cultured in T75 flasks in DMEM (Sigma-Aldrich) and RPMI-1640 medium (Sigma-Aldrich), respectively, supplemented with 10% FBS, 1 mM sodium pyruvate, and Pen Strep (all from Thermo Fisher Scientific). For hypoxia treatment, 100000 A549 or 200000 MCF-7 cells were seeded in 12-well plates. Twenty-four hours after seeding, cells were exposed to hypoxia (0.5% O_2_, 37°C, 5% CO_2_) for 48 h.

### RNA preparation and sequencing

For RNA-Seq, total RNA was isolated using the miRNeasy Mini kit (Qiagen), including the optional on-column DNA digestion with the RNase-Free DNase Set (Qiagen). After isolation, 500 ng RNA were quality checked on a 1% agarose gel. rRNA was depleted using the RiboZero kit (Zymo). Libraries were prepared and sequenced on a Illumina NextSeq sequencer with HighOutput (75-nt single-end reads) obtaining ~100 Mio reads per sample. For HeLa cells, two and three biological replicates were prepared for the hypoxic and normoxic conditions, respectively. For A549 and MCF-7 cells, two biological replicates were prepared for each cell line and condition.

### Processing of sequencing reads and genomic mapping

Quality checks were performed to all sequenced reads using FastQC (https://www.bioinformatics.babraham.ac.uk/projects/fastqc/). Reads were filtered based on sequencing quality (Phred score > 20) and read length (>20 nt) using Flexbar (version 2.5; Dodt et al., 2012).

For each sample, filtered reads were mapped to the human genome (version hg38/GRCh38), based on GENCODE reference annotation (release 24). For differential gene expression analysis and circRNA quantification (see below), mapping was performed using STAR version 2.4.5a (Dobin et al., 2012), a splice-aware mapper capable of predicting so-called chimeric alignments, i.e. discontinuous alignments in which two fragments of the read align in a non-linear order (including head-to-tail arrangements). We allowed up to two mismatches and kept only uniquely mapped reads for downstream analysis. In order to detect chimeric reads, we set the following parameters: −−chimSegmentMin 15 −−chimScoreMin 15 −−chimScoreSeparation 10 −−chimJunctionOverhangMin 15. For circRNA quantification using find_circ, the same reads were also mapped using Bowtie2 as described in (Memczak et al., 2013) using the parameter  −−score-min=C,–15,0.

### Identification and quantification of human circRNAs

To comprehensively identify circRNAs from the RNA-Seq data, sequencing reads of the different conditions were merged into a single fastq file and mapped to the human genome (version GRCh38/hg38) with Bowtie2 (Langmead and Salzberg, 2012) and STAR (Dobin et al., 2012), as described above. Unmapped reads from Bowtie2 were used to detect circRNAs with find_circ as described in (Memczak et al., 2013). Chimeric junctions reported by STAR were used to detect circRNAs with CIRCexplorer as described in (Zhang et al., 2014). In the next step, we combined the circRNAs identified by either algorithm and then systematically filtered out detection artifacts. In particular, we kept only circRNAs with a genomic distance between back-splice < 100 kb and a GU/AG or GC/AG pairing of splice site motifs, and excluded circRNAs spanning multiple non-overlapping genes. We used a custom script (R version 3.4.3) to obtain unified read count estimates from the predicted chimeric alignments by STAR for each sample, discriminating putative PCR artifacts based on the mapping position rather than read sequence. To detect a circRNA as present in a given cell line, we demanded a minimum of two distinct reads supporting the back-splice junction in at least one sample (for further details, see Supplementary Note).

### Expression analysis of circRNAs and host genes

To estimate the expression value of circRNAs (Figure 3B), we normalized the number of back-splice reads (unique and non-unique) per million mapped reads as ‘reads per million’ (RPM). To evaluate the expression level of the host gene, we used the tool htseq-count (Anders et al., 2015) to count overlapping reads per gene using default parameters. Then, we estimated the relative expression level of the host gene in TPM using custom scripts. In order to avoid bias introduced by the associated circRNA produced from the same gene, we omitted reads from exons that were predicted to be internal to the circRNAs.

In order to estimate the back-splicing rate, we count back-splice reads supporting a given circRNA as well as reads supporting linear junctions from the same splice sites (linear-junction reads), taking the mean count of linear junctions from either splice site. For the CLR (Figure 4D; Supplementary Figure S4D and E), we divided the back-splice reads by the linear-junction reads after adding a pseudocount of 1. For the ‘percent circularized’ metric (Figure 3D), we divided the back-splice reads by the sum of back-splice and linear-junction reads, multiplied by 100. We note that both metrics can be flawed when the circRNAs include cryptic back-splice sites that are not activated under normal conditions, such as exon 1A in circZNF292 (intronic; Boeckel et al., 2015). Moreover, the metric currently ignores isoforms which completely skip the involved exon(s), which can result in an overestimation of the relative abundance of the circRNA isoform.

To evaluate the differential expression and alternative splicing of genes between normoxia and hypoxia in the three cell lines (Supplementary Figure S1), we used DESeq2 version 1.18 (Love et al., 2014), setting an adjusted *P*-value threshold of 0.05. The analysis was restricted to genes with a total of at least 10 reads (raw counts) in the tested cell line. To detect alternative splicing events, we used rMATS version 3.2.5 (Shen et al., 2014) with parameter –*c* 0.0001, considered reads mapped to splice junctions, setting a cutoff at a FDR < 0.05 and absolute change in ‘percent spliced in’ (|ΔPSI|) ≥ 10%.

To evaluate the differential expression of circRNAs (Figure 4A; Supplementary Figure S4A), we relied on back-splice reads and DESeq2 (Love et al., 2014) to identify significant differences (FDR < 0.1). For this analysis, the raw back-splice read counts of the circRNAs were combined with the raw read counts on the complete genes to improve library size estimation, normalization, and statistical power.

### RNA preparation and RT-(q)PCR for validation experiments

HeLa and MCF-7 cells were cultured and exposed to hypoxia as described above. After hypoxia treatment, cells were harvested and resuspended in Trizol for RNA extraction, followed by DNase treatment (Turbo DNase, Invitrogen).

For validation of circularity, two approaches were taken, based on (i) polyA(+) RNA separation and (ii) RNase R treatment. All validation experiments were performed with a representative sample of HeLa cells under normoxic conditions. PolyA(+) RNA separation was performed using Oligo d(T)25 magnetic beads (New England Biolabs) following the manufacturer’s protocol with small modifications. Briefly, 10 μg total RNA were incubated with 50 μl beads. The supernatant was collected and saved as polyA(−) fraction. To achieve higher purity, the polyA(−) fraction was incubated a second time with fresh beads, and the protocol repeated. For the bead-bound polyA(+) fractions, protocol and washes were continued as recommended by the manufacturer. After washing, eluted polyA(+) RNA and polyA(−) RNA suspension were precipitated overnight by adding ethanol to a final concentration (f.c.) of 70% and sodium acetate (0.3 M f.c.). For the RNase R treatment, 10 μg total RNA were incubated at 37°C for 40 min with or without 10 units of RNase R (Epicenter), followed by 3 min incubation at 95°C for RNase R inactivation. The reaction was performed in 20 μl. After treatment, ethanol (70% f.c.) and sodium acetate (0.3 M f.c.) were added for precipitation.

After treatment and precipitation, RNA was recovered and cDNAs were synthetized by RT-PCR using SuperScript III Reverse Transcriptase (Life Technologies), dNTPs, and random hexamers (dNTP Mix and Hexanucleotide Mix, Sigma-Aldrich) following the SuperScript III protocol recommended by the manufacturer. The presence of the circRNAs specifically in the polyA(−) fraction and the RNase R-treated samples was confirmed using divergent primers flanking the back-splice junctions (primers were designed using SnapGene and ordered at Sigma-Aldrich) by semi-quantitative PCR. Primers against linear *PLOD2* mRNA were used as control. The PCR reaction was performed with Phusion Polymerase (New England Biolabs), 10 mM dNTPs (dNTP Mix, Sigma-Aldrich), 10 nM forward and reverse primers, and 1:1 DMSO per Phusion volume. After preparing the master mix, 1 μl cDNA was added to the reaction, and the PCR was performed in the following conditions: 98°C for 2 min, 34 cycles of 98°C for 30 sec, 55°C–60°C (depending on the primer) for 30 sec, and 72°C for 30 sec, and final extension at 72°C for 5 min. PCR products were visualized using 2% agarose gel electrophoresis (VWR Maxi or Midi Electrophoresis System). The 2% agarose gels were pre-stained with RedSafe (HiSS Diagnostics).

For validation of differentially expressed circRNAs under hypoxia, RNA was prepared from hypoxic and normoxic HeLa and MCF-7 cells, and reverse transcription was performed from 2 μg total RNA as described above. Differential expression was validated by RT-qPCR using 1× final concentration of 2X ORA qPCR Green ROX L Mix (highQu GmbH), 500–2000 nM forward and reverse primers (depending on the primer) and 1 μl of 1:8 dilution of cDNA. Primers targeting U6 for HeLa and P0 for MCF-7 were included in each experiment, and its quantification cycle number (*C_q_* value) posteriorly used for normalization. The RT-qPCR was performed in a PikoReal 96 Real-Time PCR System (Thermo Fisher Scientific) using the following program: 95°C for 2 min, 30 cycles of 95°C for 20 sec, 60°C for 20 sec, and 72°C for 30 sec, and final extension at 72°C for 5 min, followed by a step-wise melting curve (60°C–95°C). The same primers were used for semi-quantitative PCR and RT-qPCR (Supplementary Table S4).

### Genomic annotation and molecular characterization of circRNAs

We used custom scripts (R version 3.4.3) to compare the genomic coordinates of the circRNAs to genomic/transcript features taken from GENCODE reference annotation (release 24, basic annotation). Only genes with support level 1 or 2 were considered, thus discarding automatically annotated genes. In order to assign exon ranks and possible internal exons of the circRNAs, we relied on canonical splice variants (knownCanonical, downloaded from UCSC Genome Browser) where possible. If back-splice junctions did not match a canonical transcript, we searched for non-canonical transcripts with coincident splice sites, thus defining a best parental transcript. For the internal structure of the circRNA, we conservatively assumed that all annotated exons of the parental transcript that lay between the back-splice sites were included into the circRNA. If no annotated exons overlapped with a circRNA, it was considered as intronic/intergenic depending on its genomic location. circRNA overlapping with >1 annotated gene were labeled as ‘ambiguous’. Finally, we further excluded putative back-splicing events spanning multiple non-overlapping genes (*n* = 70) for downstream analysis.

Splice site strengths were predicted using the sequence analysis software MaxEntScan (Yeo and Burge, 2004; Figure 2C). GO-enrichment analysis was performed using the overrepresentation test implemented in clusterProfiler package version 3.6.0 (Yu et al., 2012) in the R statistical software environment (version 3.4.3). Enrichment was tested against the union of all genes that were tested in the DESeq2 analysis of any cell line. *P*-value and *q*-value cutoffs were set to 0.05. Biological process and molecular function categories were explored.

*Alu* elements in flanking introns were analyzed based on RepeatMasker annotation (www.repeatmasker.org) downloaded from UCSC Genome Browser. For the 64 hypoxia-regulated circRNAs, we additionally performed pairwise local alignments of sequences in a 500-bp window up- and downstream of the back-splice sites using the R package Biostrings (version 2.46.0; parameters gapOpening = 10 and gapExtension = 4). The difference in the association of regulated (Reg) vs. all circRNA with *Alu* elements (Figure 5B) did not reach statistical significance in a *Chi*-squared test (*P*-value > 0.1).

### Prediction of miRNA target sites and RBP binding sites

In order to predict potential interactions between circRNAs and miRNAs, the sequences and annotation of a high-confidence set of 542 miRNAs in humans (high_conf_mature.fa.gz) were obtained from the miRBase database (http://www.mirbase.org/). Next, we used miRanda (version 3.3a) to predict miRNA target sites on circRNAs, setting match score >150 and using the parameter strict to demand a strict alignment in the seed region. This yielded a set of high-confidence miRNA target sites. The analysis was performed on a subset of 9754 circRNAs (only high-confidence circRNAs are shown in the figures), for which we could assign a parental transcript as described above. The previously published, intronic isoform of circZNF292 (hsa_circ_0004383) was manually added to the list (Boeckel et al., 2015; excluding the cryptic intron between exons 1A and 2). As a control, we included the circRNA CDR1as/ciRS-7 (Hansen et al., 2013; Memczak et al., 2013). The frequency of miRNA target sites within the circRNA sequences was compared to 10000 CDS and 3′ UTR sequences that randomly selected from the GENCODE annotation. The number of detected targets sites in a circRNA, CDS, or 3′ UTR was normalized by the size of the region (Supplementary Figure S5F).

Putative RNA binding sites were searched in regions flanking the 64 hypoxia-regulated circRNAs as well as the 2141 unchanged circRNAs from the high confidence set, considering a 1000-bp window up- and downstream of the back-splice sites. We used FIMO (version 5.0.2; Grant et al., 2011), a program integrated in the MEME suite (http://meme-suite.org/), to search for known RBP motifs from *in vitro* binding assays (Ray et al., 2013) downloaded from the MEME database (Ray2013_rbp_Homo_sapiens.dna_encoded.meme). A *P*-value cutoff of 0.0001 was applied. Only predicted binding sites with *q*-value < 0.05 were considered for downstream analysis.

### HNRNPC iCLIP data analysis

To verify the binding of HNRNPC to regions flanking circRNAs, we analyzed HNRNPC iCLIP data in HeLa cells from Zarnack et al. (2013). Peak calling was performed on merged iCLIP tracks with PureCLIP version 1.1.2 (Krakau et al., 2017) setting the parameter -ld to use higher precision to compute emission probabilities. Crosslink sites were merged when located at a distance ≤8 nt. To investigate the spatial distribution of HNRNPC binding to regions flanking circRNAs, the average read coverage at each position was calculated considering an intronic window of 1000 nt up-/downstream of the back-splice sites plus the first/last 50 nt of the circularized exon(s). A total of 1133 high-confidence circRNAs expressed in HeLa cells and originated from PCGs were investigated, separated into hypoxia-regulated and non-regulated circRNAs. HNRNPC binding to circularizing exons was compared to linear control exons from expressed PCGs that do not undergo circularization. Similar to the criteria for the high-confidence set of circRNAs, only internal, linearly spliced exons with a minimum of five inclusion junction reads in any two samples in HeLa were used as control exons in this comparison (Figure 5E).

### siRNA transfection for HNRNPC knockdown

*HNRNPC* KD was performed using previously described siRNAs (Zarnack et al., 2013): Stealth Select RNAi siRNAs HSS179304 and HSS179305 as well as control siRNA Stealth RNAi siRNA Negative Control. For KD experiments, HeLa cells were cultured under normal conditions and seeded into 6-cm dishes 24 h prior to siRNA transfection. A final concentration of 20 nM of each siRNA was transfected into HeLa cells using jetPRIME^®^ DNA and siRNA transfection reagent (VWR) following the manufacturer’s protocol. KD was performed for 48 h, and cells were subsequently harvested for RNA extraction as described above. The functional KD was confirmed by measuring the splicing changes in four known target exons from HNRNPC (Zarnack et al., 2013).

### Data availability

The RNA-Seq data from this study have been submitted to the Gene Expression Omnibus (GEO; www.ncbi.nlm.nih.gov/geo/) with accession number GSE131379.

## Supplementary Material

Supplementary_material_mjz094

Supplementary_Table_2_circRNA_catalogue_mjz094

Supplementary_Table_3_hypoxia_regulated_circRNAs_mjz094
