# Supplementary material for: A combined computational pipeline to detect circular RNAs in human cancer cells under hypoxic stress
Source: J Mol Cell Biol. 2019 Sep 27;11(10):829–44. doi: 10.1093/jmcb/mjz094 (PMC6884703; doi:10.1093/jmcb/mjz094)
Supplement: Supplementary_material_mjz094 [file supplementary_material_mjz094.pdf]

A combined computational pipeline to detect circular RNAs in human cancer cells under hypoxic stress

Antonella Di Liddo[1,2], Camila de Oliveira Freitas Machado[2,3], Sandra Fischer[4], Stefanie Ebersberger[5], Andreas W. Heumüller[3], Julia E. Weigand[4], Michaela Müller-McNicoll[2], Kathi Zarnack[1]

[1] Buchmann Institute for Molecular Life Sciences, Goethe University, Frankfurt am Main, Germany; [2] Institute of Cell Biology and Neuroscience, Goethe University, Frankfurt am Main, Germany; [3] Institute for Cardiovascular Regeneration, Goethe University, Frankfurt am Main, Germany; [4] Department of Biology, Technical University Darmstadt, Germany; [5] Institute of Molecular Biology (IMB), Mainz, Germany. email: kathi.zarnack@bmls.de

SUPPLEMENTARY MATERIAL

Content:

Supplementary Note 1..2

Supplementary References..6

Supplementary Tables 1 & 4..7

Supplementary Figures 1-5..10

Supplementary Note 1

Consolidation of approaches to identify circRNAs from rRNA-depleted RNA-Seq data

Several computational tools are available to detect circRNAs from RNA-Seq data (reviewed in (Szabo and Salzman, 2016; Gao and Zhao, 2018). Two recent studies reported a small overlap between predictions when different tools were benchmarked on the same datasets (Hansen et al., 2015; Zeng et al., 2017), suggesting that a combination of multiple algorithms is advisable to obtain a more reliable list of candidate circRNAs. In this study, we sought to apply two established algorithms, find_circ (Memczak et al., 2013) and CIRCexplorer (Zhang et al., 2014) in order to comprehensively profile the circRNA repertoire of different cancer cell lines.

We extensively characterized the predicted circRNA sets to identify the specific strengths and weaknesses of either algorithm. In particular, we tested the different algorithms by find_circ and CIRCexplorer on a single sample from HeLa RNA-Seq data. As described in (Memczak et al., 2013), we filtered find_circ output demanding unique alignments, unambiguous breakpoint, GU/AG splice site signal and a maximum genomic distance of 100 kb between the back-splice sites, obtaining a total of 3,752 circRNAs. Then, we applied a further filter on counted reads, demanding a minimum of two distinct reads supporting the back-splice sites, thus reducing the number of circRNAs to 1,118. Only 37% of them could be detected in at least two replicates with the same algorithm, suggesting a certain level of noise in the find_circ predictions. This is also reflected in a larger fraction of false-positive predictions (see below, **Supplementary Figure 2F**). The CIRCexplorer output included 3,343 circRNAs, that were further filtered demanding a minimum of two reads supporting the back-splice sites, to get a final list of 2,269 circRNAs (2,193 exonic circRNAs, 76 ciRNAs). 68% of them could be detected in at least one of the other replicates with the same algorithm.

When we compared the final outcomes of find_circ and CIRCexplorer, the two algorithms agreed on the prediction of 903 circRNAs, while further 285 and 1,366 circRNAs were reported exclusively by find_circ and CIRCexplorer, respectively (**Supplementary Figure 2A**). In addition, for circRNAs detected by both tools, the detected number of reads spanning the back-splicing junctions was highly correlated, but not completely consistent (**Supplementary Figure 2B**).

Next, we investigated the underlying reasons of the discrepancy in the amount of detected circRNAs. Examining the number of reads supporting the back-splicing junctions, circRNAs

detected by a single tool were significantly less abundant than circRNAs identified by both algorithms. However, several circRNAs resulted to be particularly abundant (**Supplementary Figure 2C**). First, we looked at circRNAs detected only by find_circ (n = 285), finding that 191 (67%) were included in the chimeric alignments by STAR but not reported in CIRCexplorer output, mostly because they originated from unannotated junctions (n = 133).

Then, we investigated circRNAs detected only by CIRCexplorer (n = 1,366) and found that 26 (2%) circRNAs spanned a genomic region larger than 100 kb. Moreover, 24 of them were initially detected by find_circ but removed by the genomic distance filter. We also noticed that 85 circRNAs (6%) showed noncanonical splice site motifs, even though they originated from annotated splice sites. Finally, albeit HeLa cells were taken from a female patient, four circRNAs were annotated on chromosome Y. These circRNAs resided in the pseudoautosomal regions (PAR) which are homologous between chromosomes X and Y.

While the above-mentioned filters only slightly influenced the overlap between results from find_circ and CIRCexplorer, we found that the threshold placed on supporting back-splice reads drastically impacted on the final outcome. find_circ provides two different measures of supporting back-splice reads, counting either all back-splice reads, thus including PCR duplicates, or just unique back-splice reads, which are distinct in their sequence and hence likely arise from independent reverse transcription events. find_circ's expression filter is based on unique back-splice reads to avoid putative PCR artifacts. On the opposite, CIRCexplorer does not count unique back-splice reads. As a consequence, 68% (n = 936) of the CIRC-explorer-only circRNAs were also initially reported by find_circ first but then excluded as putative PCR duplicates as they were supported by only a single unique back-splice read. Consistently, when we compared the CIRCexplorer output to circRNAs found with find_circ when demanding a minimum of two any reads, independently on their sequence (n = 2,513), the overlap between both tools largely increased.

Based on these results, we established a new pipeline to unify the results of both tools and thus obtain a comprehensive catalogue of circRNAs from our RNA-Seq datasets (**Figure 1A**). For each cell type, sequencing reads of the different conditions were merged and mapped to the human genome (version GRCh38/hg38) with Bowtie2 (Langmead and Salzberg, 2012) and STAR (Dobin et al., 2012). Unmapped reads from Bowtie2 were used to detect circRNAs with find_circ as described in (Memczak et al., 2013). Chimeric junctions resulting from STAR alignment were fed into CIRCexplorer. Although keeping only circRNAs that are detected by both tools would increase the reliability of the candidates, this would leave out

abundant circRNAs that are missed by one of the two algorithms due to its specific criteria. For instance, the abundant and hypoxia-regulated circZNF292 (Boeckel et al., 2015) originates from a back-splice site within an intron, and it is thus not identifiable by CIRCexplorer. In a next step, we united the circRNAs identified by either algorithm and then systematically filtered out the detection artifacts described above. In particular, we removed circRNAs with a genomic distance between back-splice sites of more than 100 kb. Regarding the sequence at predicted back-splice sites, we kept circRNAs with canonical splice site motifs (GU/AG) as well as with GC/AG pairs, which represent the most frequent noncanonical splice site motifs (Burset et al., 2000). We finally excluded circRNAs with back-splice junctions that were predicted to reside in non-overlapping genes. For the three analyzed cancer cell lines, this procedure yielded a total of 12,006 predicted circRNAs.

In order to obtain consistent back-splice read count estimates, we used a custom script to recount back-splice reads from STAR's chimeric alignments. We discriminated unique back-splice reads from putative PCR artifacts based on the mapping position rather than read sequence. To detect a circRNA as present in a given cell line, we demanded a minimum of two unique back-splice reads supporting the back-splice junction in at least one sample. For the abundance estimates, unique and non-unique back-splice reads were taken into account.

In order to evaluate the sensitivity and specificity of our pipeline, we tested it on a published dataset from human Hs68 cells, in which circRNAs had been specifically enriched by RNase R digestion followed by RNA-Seq (Jeck et al., 2013). Similar to previous publications (Hansen et al., 2015; Wang et al., 2017; Zeng et al., 2017; Hansen, 2018), we predicted circRNAs in the rRNA-depleted total RNA-Seq data ("total") and compared their back-splice read counts to the levels in the RNase R-treated RNA-Seq data ("RNase R"). In order to compare the performance of our pipeline to the isolated tools, we also ran CIRCexplorer and find_circ separately. For comparability, we applied the same filter criteria for both tools on supporting back-splice reads, i.e. at least two supporting back-splice reads (unique or non-unique) in one of the total RNA samples to keep a circRNA prediction, following the standard CIRCexplorer approach. For find_circ, we additionally tested the detection performance requiring at least two unique supporting back-splice reads in one of the total RNA samples, as suggested in the original publication (Memczak et al., 2013) and proposed in our combined pipeline. For quantification of the circRNAs, raw back-splice read counts were normalized to sequencing depth, and replicates were combined by averaging the normalized counts. As in the previous publications, the derived fold-enrichment values ($ratio = RNase\ R \div total$)

were subdivided into depleted and RNase R-resistant circRNAs ($ratio < or > 1$, respectively). These were further stratified into <5-fold reduction ($ratio < 0.2$; strong depletion), 5-1-fold reduction ($ratio \in [0.2; 1]$; modest depletion), 1-5-fold increase ($ratio \in [1; 5]$; modest enrichment), and >5-fold increase ($ratio > 5$; strong enrichment). RNase R-resistant circRNAs were considered as true-positives in the following evaluation. We found that our pipeline detected the RNase R-resistant circRNAs with 80% precision (**Supplementary Figure 2F**). The precision was higher than for find_circ with either settings (66% and 70%) and comparable to CIRCexplorer (80%). Individually run, both tools predicted more circRNAs, reflected in slightly higher absolute numbers of true- and false-positives. In the case of CIRCexplorer, the higher numbers most likely result from the more lenient requirement of any two back-splice reads (compared at least two unique back-splice reads in our pipeline). In essence, our pipeline combines reliable circRNA predictions from both tools, thereby extending the scope of CIRCexplorer to intronic back-splice sites and higher stringency on unique back-splice reads, while maintaining high precision. Altogether, we conclude that our combined pipeline is well suited to reliably predict circRNAs with high precision.

Supplementary References

Boeckel, J.-N., Jaé, N., Heumüller, A.W., et al. (2015). Identification and Characterization of Hypoxia-Regulated Endothelial Circular RNA. Circ Res *117*, 884–890.

Burset, M., Seledtsov, I.A., and Solovyev, V.V. (2000). Analysis of canonical and non-canonical splice sites in mammalian genomes. Nucleic Acids Res *28*, 4364–4375.

Dobin, A., Davis, C.A., Schlesinger, F., et al. (2012). STAR: ultrafast universal RNA-seq aligner. Bioinformatics *29*, 15–21.

Gao, Y., and Zhao, F. (2018). Computational Strategies for Exploring Circular RNAs. Trends Genet *34*, 389-400.

Hansen, T.B. (2018). Improved circRNA Identification by Combining Prediction Algorithms. Front Cell Dev Biol *6*, 20.

Hansen, T.B., Venø, M.T., Damgaard, C.K., et al. (2015). Comparison of circular RNA prediction tools. Nucleic Acids Res *44*, e58.

Jeck, W.R., Sorrentino, J.A., Wang, K., et al. (2013). Circular RNAs are abundant, conserved, and associated with ALU repeats. RNA *19*, 141–157.

Langmead, B., and Salzberg, S.L. (2012). Fast gapped-read alignment with Bowtie 2. Nat Meth *9*, 357–359.

Memczak, S., Jens, M., Elefsinioti, A., et al. (2013). Circular RNAs are a large class of animal RNAs with regulatory potency. Nature *495*, 333–338.

Szabo, L., and Salzman, J. (2016). Detecting circular RNAs: bioinformatic and experimental challenges. Nat Rev Genet *17*, 679–692.

Wang, J., Liu, K., Liu, Y., et al. (2017). Evaluating the bias of circRNA predictions from total RNA-Seq data. Oncotarget *8*, 110914–110921.

Zeng, X., Lin, W., Guo, M., et al. (2017). A comprehensive overview and evaluation of circular RNA detection tools. PLoS Comput Biol *13*, e1005420–e1005421.

Zhang, X.-O., Wang, H.-B., Zhang, Y., et al. (2014). Complementary Sequence-Mediated Exon Circularization. Cell *159*, 134–147.

Supplementary Tables

Supplementary Table 1. Overview of RNA-Seq datasets used in this study. Table includes information of cell line, condition (N, normoxia; H, hypoxia) and replicate (1, 2, 3), alignment statistics for each dataset and circRNA identification.

Cell line	Sample	Total reads	rRNA reads (%)	Processed reads	Mapped reads (%)	Back-splice reads per million mapped	# circRNAs	Total
**A549**	N1	118,103,929	8.07	117,993,957	78.91	144	2,870	**4,599**
**A549**	N2	143,641,614	8.51	143,504,453	77.65	129	2,998	
**A549**	H1	105,410,274	5.34	105,296,954	79.46	186	1,999	
**A549**	H2	121,800,191	4.34	121,681,536	81.06	141	2,406	
**HeLa**	N1	60,631,903	4.07	60,595,974	85.77	134	1,508	**3,926**
**HeLa**	N2	64,227,967	3.72	64,188,177	86.69	116	1,489	
**HeLa**	N3	62,772,278	4.07	62,733,339	85.20	155	1,728	
**HeLa**	H1	64,106,501	4.22	64,067,445	84.81	154	1,746	
**HeLa**	H2	66,800,563	3.58	66,759,182	85.27	169	1,973	
**MCF-7**	N1	113,507,866	2.32	113,397,461	81.51	242	4,194	**7,527**
**MCF-7**	N2	113,938,974	2.13	113,825,152	82.01	282	4,275	
**MCF-7**	H1	113,450,829	2.66	113,340,824	81.27	325	4,612	
**MCF-7**	H2	125,385,855	9.28	125,263,956	73.68	382	5,429	

Supplementary Table 4. Oligonucleotides used in this study.

Target region	Primer name	Primer sequence (5´->3´)	Amplicon size (bp)
**circRNAs**			
circGSE1	hcirc_GSE1_fw	CCAGCTTTGCCGCCGCGCTG	61
	hcirc_GSE1_rw	GTGGAAAGCATCCCTAGCG	
circHIPK3	hcirc_HIPK3_fw	TCGGCCAGTCATGTATCAAA	196
	hcirc_HIPK3_rw	CCCTTAGTGGGAGGATGAGA	
circCAMSAP1	hcirc_CAMSAP1_fw	CCCTGATGATGGCCTACACT	157
	hcirc_CAMSAP1_rw	TGTGCTCCTGCTCATACTGG	
circRICTOR	hcirc_RICTOR_fw	GAAAGAGACAGAATGGTCCGAGC	230
	hcirc_RICTOR_rw	ACCTCGTTGCTCTGTTGTATGTC	
circREV1	hcirc_REV1_fw	AGTTTCGATCAGATGCTGCTATGC	128
	hcirc_REV1_rw	ACCTTGGCAGCCATATACCCAC	
circGCN1	hcirc_GCN1_fw	GTGCTGGATGCTTTGGGACG	201
	hcirc_GCN1_rw	TCAATCAGCAAGGAGCAGAGGTC	
circSLTM	hcirc_SLTM_fw	AAGAGGACATCGAAAGTCAGG	118
	hcirc_SLTM_rw	GTGCCTCTTGATTCTCCAATTC	
circAAGAB	hcirc_AAGAB_fw	GAGGAGTTGCCTGAGGAGGATG	116
	hcirc_AAGAB_rw	TCTCACAGCATCATTGGAAGTCAC	
circPLOD2	hcirc_PLOD2_fw	GAAGTCATGGAACACTATGCTG	132
	hcirc_PLOD2_rw	ACCTCTCCATTCTTCTCCTTGACC	
circMAN1A2	hcirc_MAN1A2_2_fw	GGGCAAAGATGGATTGAAGACAACC	167
	hcirc_MAN1A2_2_rw	TTGCTTCTTCCAAGGCCTTCTCATG	
circZNF292 (exonic)	hcirc_exonicZNF292_fw	ACCCGGTACTGTGCACTATTCTTTC	151
	hcirc_exonicZNF292_rw	GGTCGGGCTTTAACATAACTTTGG	
circZNF292 (intronic)	hcirc_intronicZNF292_fw	GCTCAAGAGACTGGGGTGTG	97
	hcirc_intronicZNF292_rw	AGTGTGTGTTCTGGGGCAAG	
circSRSF4	hcirc_SRSF4_fw	AAGACAAGCCAGGTTCCAGA	116
	hcirc_SRSF4_rw	TTTTGCGTCCCTTGTGAGC	
circCDYL	hcirc_CDYL_fw	CAGGCTTAGCTGTTAACGGG	60
	hcirc_CDYL_rw	TGTCATAGCCTTTCCACCGA	
circATXN7	hcircATXN7_fw	GCCCGCTCCGACATTCTTTC	137
	hcircATXN7_rw	TGGAATCTGTGGGTTGAGGCTTC	
circCPSF6	hcircCPSF6_fw	TATTACAGAGAGAGAAGCAGAGAACGAGAGAG	245
	hcircCPSF6_rw	GCTCCTTTACCCACATCATCACCAACAG	
circHERC4	hcircHERC4_fw	TGGAGGGTATGGTCAGTTGG	208
	hcircHERC4_rw	AGACCCCAAGCATACACCTG	
circCDYL2	hcircCDYL2 _fw	AGGACTACCCCCAGTGGTTT	213
	hcircCDYL2 _rw	CTTTCAACCGAGCCCGTTCT	
circCLASP1	hcircCLASP1_fw	GCAATTACAAGAATGGACTGCTGA	172
	hcircCLASP1_rw	ATGCAATCTGGGGAATGGCA	
circRARS	hcircRARS_fw	CCAGTTTGGCATGCTCATCG	151
	hcircRARS_rw	ACAGCCACAGTTTTTCAACCG	
circRERE	hcircRERE_fw	CCCTGAGACAAGAGTAAGAGGG	105
	hcircRERE_rw	TCGATCCTGAACCAAATGCTGA	

(continued on next page)

Supplementary Table 4. Oligonucleotides used in this study (continued).

Target region	Primer name	Primer sequence (5´->3´)	Amplicon size (bp)
**circRNAs**			
circSTX6	hcircSTX6_fw	TGACCAGTGGACATGAAAGATCA	119
	hcircSTX6_rw	GTTGTTCCAGTGCTCCAGTTC	
circANKRD12	hcircANKRD12_fw	TAAACATGGGGAGCGTCCAG	172
	hcircANKRD12_rw	GTGAACCCAGATTTGGGCATT	
circPHC3	circPHC3_fw	GTACAGTCTGACATTCCTGTTGTCT	152
	circPHC3_rw	TGTTGGGTAATACTGCCGCTGG	
circSMARCA5	circSMARCA5_fw	CTCCAAGATGGGCGAAAG	173
	circSMARCA5_rw	TGTGTTGCTCCATGTCTAATCA	
circMTCL1	circMTCL1_fw	GCTTATTCGAAGCCTGGAGCAG	147
	circMTCL1_rw	CTCTAAATAACTGTCTCTCATCTCTTCCATCTC	
circRTN4	circRTN4_fw	TCTTATTGCCTCCAGATGTTTCTGCTTT	223
	circRTN4_rw	GCAGAGGAGCGTATCACAGG	
circSPECC1	circSPECC1_fw	AAATGTTGAAAGTAGCCCGAGCAG	162
	circSPECC1_rw	CGTGGGGCTGGAATGCC	
**Linear transcripts**			
linear *VEGFA* mRNA	hVEGFA-fw	ATCTGCATGGTGATGTTGGA	218
	hVEGFA-rw	GGGCAGAATCATCACGAAGT	
linear *PLOD2* mRNA	hLin_PLOD2_fw	CTCGAGCATCCCCACAGATAAA	282
	hLin_PLOD2_rw	ACTTCTTCTGGACCACCAGC	
**HNRNPC target exons (Zarnack et al., 2013)**			
CSPP1	CSPP1_fw	GCCGAAGACAAAGCAGAGTT	77/198 (ex/in)
	CSPP1_rw	TCAGAAAGCTTCGCTGACAA	
DRAM1	DRAM1_fw	AGCCGCCTTCATTATCTCCT	94/220 (ex/in)
	DRAM1_rw	TGGAGGTGTTGTTCCCGTAT	
RAD52	RAD52_fw	CATCCAGAAGGCCCTGAG	99/230 (ex/in)
	RAD52_rw	ACCCGATGACCCTCAATGTA	
WRN	WRN_fw	TGCTAGTGATTGCTCTTTCCTG	86/156 (ex/in)
	WRN_rw	GTGGCCACTCCATGTCAAAT	

Supplementary Figures

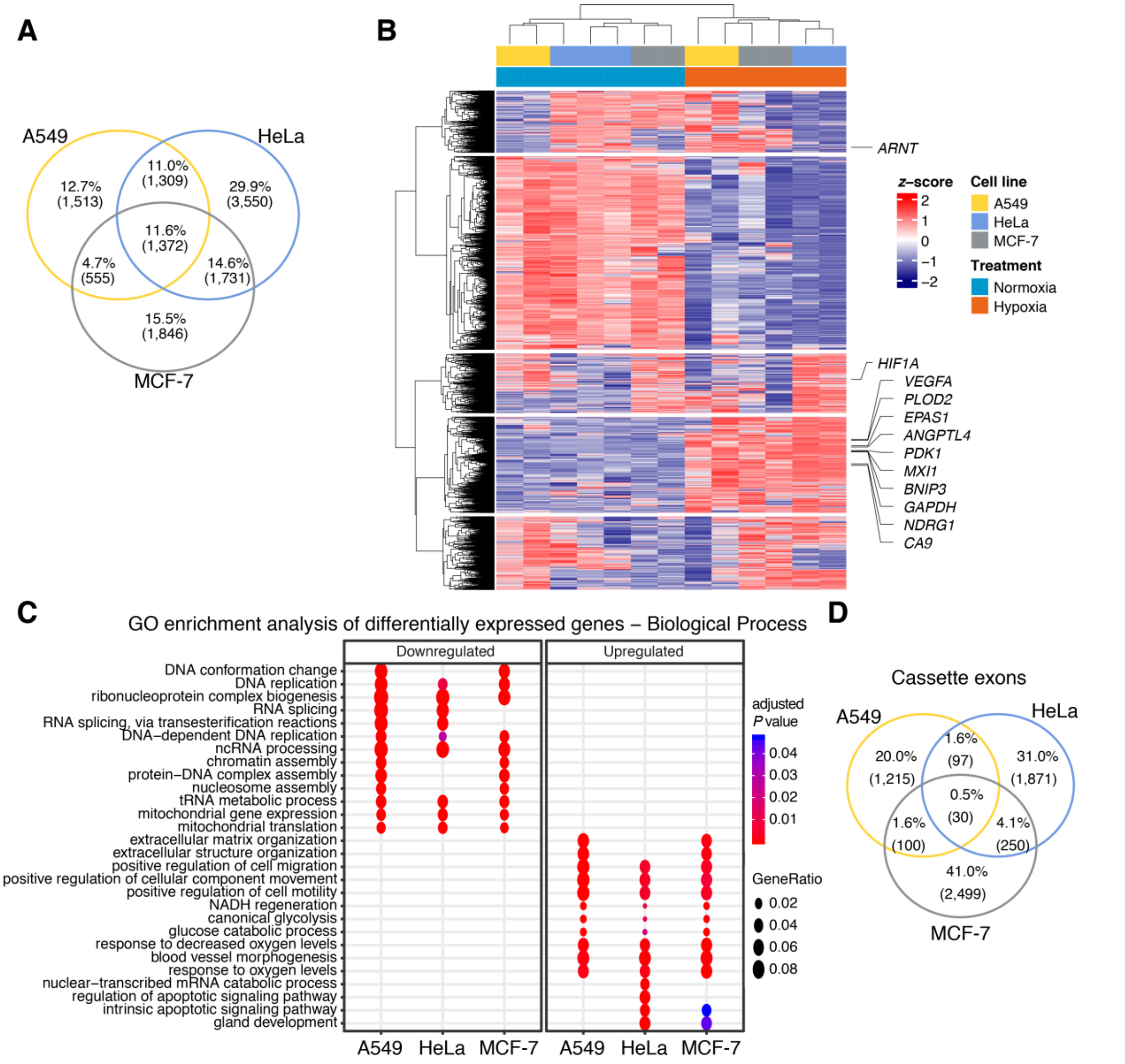

Supplementary Figure 1. Influence of hypoxia on gene expression and alternative splicing. (**A**) The three cancer cell lines show a similar response in gene expression under hypoxia. Venn diagrams show significantly differentially expressed genes between normoxic and hypoxic conditions in A549, HeLa and MCF-7 cells detected by DESeq2 (adjusted P value < 0.05). 4,976 circRNAs change their level in at least two cell lines. (**B**) Heatmap displays expression levels of 11,876 differentially expressed genes. *z*-scores were computed from log-transformed transcripts per million (TPM). Rows ordered by hierarchical clustering (based on Euclidean distance) and split into five groups based on *k*-means clustering. Several genes are labeled that are commonly upregulated under hypoxia. (**C**) Enrichment of gene ontology (GO) terms (Biological Process) for hypoxia-regulated protein-coding genes. Circle diameter corresponds to gene ratio, which reflects the number of regulated genes with a given GO term relative to the total number of regulated genes and the total number of annotated genes associated with this GO term. Circle color indicates significance (hypergeometric test

followed by Benjamini-Hochberg correction). Only GO terms with adjusted *P* value/ q value < 0.05 are shown. (**D**) Hypoxia-induced alternative splicing is highly divergent between the three cell lines. Venn diagram compares cassette exons with significant difference in inclusion upon hypoxia in the three cell lines (‘percent spliced-in’, ΔPSI > 10%, false discovery rate < 5%).

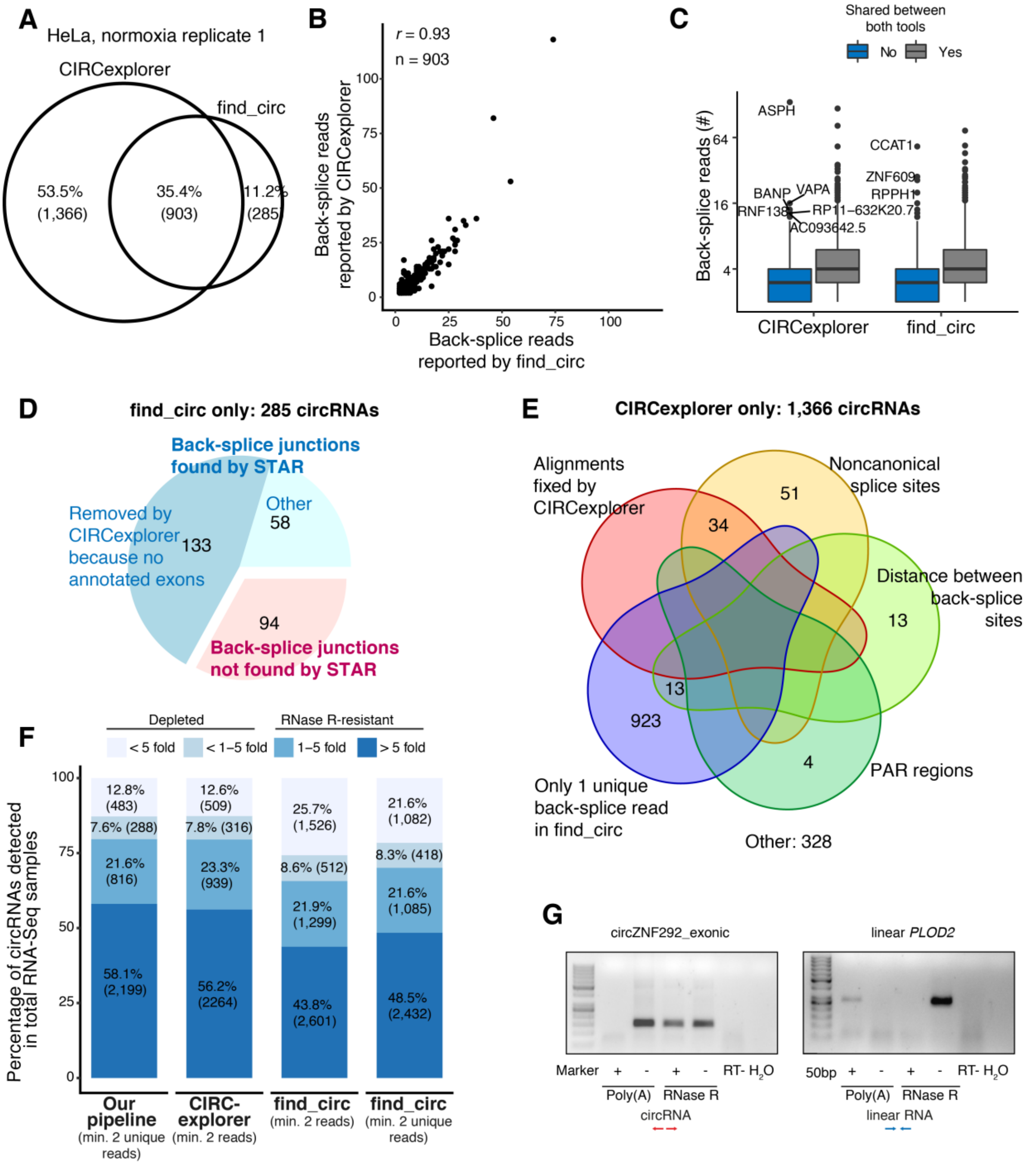

Supplementary Figure 2. Characterization of overlaps and discrepancies in circRNA predictions by find_circ and CIRCexplorer. (**A**) Venn diagram compares circRNAs predicted by CIRCexplorer and find_circ from RNA-Seq of a representative HeLa sample (normoxia, replicate 1). (**B**) Comparison of back-splice read counts for circRNAs detected by both CIRCexplorer and find_circ. (**C**) Box plot shows circRNA quantification by CIRCexplorer and find_circ, discriminating between circRNAs that are in common or predicted by only a single tool. The latter are usually supported by less back-splice reads, with notable exceptions (labeled). (**D**) Characterization of 285 circRNAs that are only predicted by find_circ (find_circ only). 33% of these circRNAs could not be detected by CIRCexplorer as the respective back-splice junctions were not present in the chimeric alignments from STAR.

(**E**) Characterization of 1,366 circRNAs that were only predicted by CIRCexplorer (CIRCexplorer only). Criteria like genomic distance of the predicted back-splice sites, splice site motifs, gene annotation, custom adjustments of alignments by CIRCexplorer and supporting back-splice reads contribute to different extent to the discrepancy between both tools. For the remaining 328 circRNAs, the reason remained unclear. Altogether, the results highlight the relevance of filtering based on unique back-splice reads, as done in find_circ pipeline. (**F**) Evaluation of circRNA predictions from our pipeline compared to CIRCexplorer and find_circ (with two settings, see Supplementary Note 1) based on published RNase R-treated RNA-Seq data. Predicted circRNAs were categorized into depleted or RNase R-resistant based on fold-enrichment of back-splice read counts in total over RNase R-treated RNA-Seq data. The pipeline performed as good as CIRCexplorer and better than find_circ, both for RNase R-resistant circRNAs in general as well as for highly enriched circRNAs (>5-fold). (**G**) Validation of circularity for the exonic ZNF292 isoform in HeLa cells, similarly to Figure 1D. RT-PCR products using divergent oligonucleotides after polyA(+) selection or RNase R treatment. Oligonucleotides amplifying the linear *PLOD2* transcript were used as control.

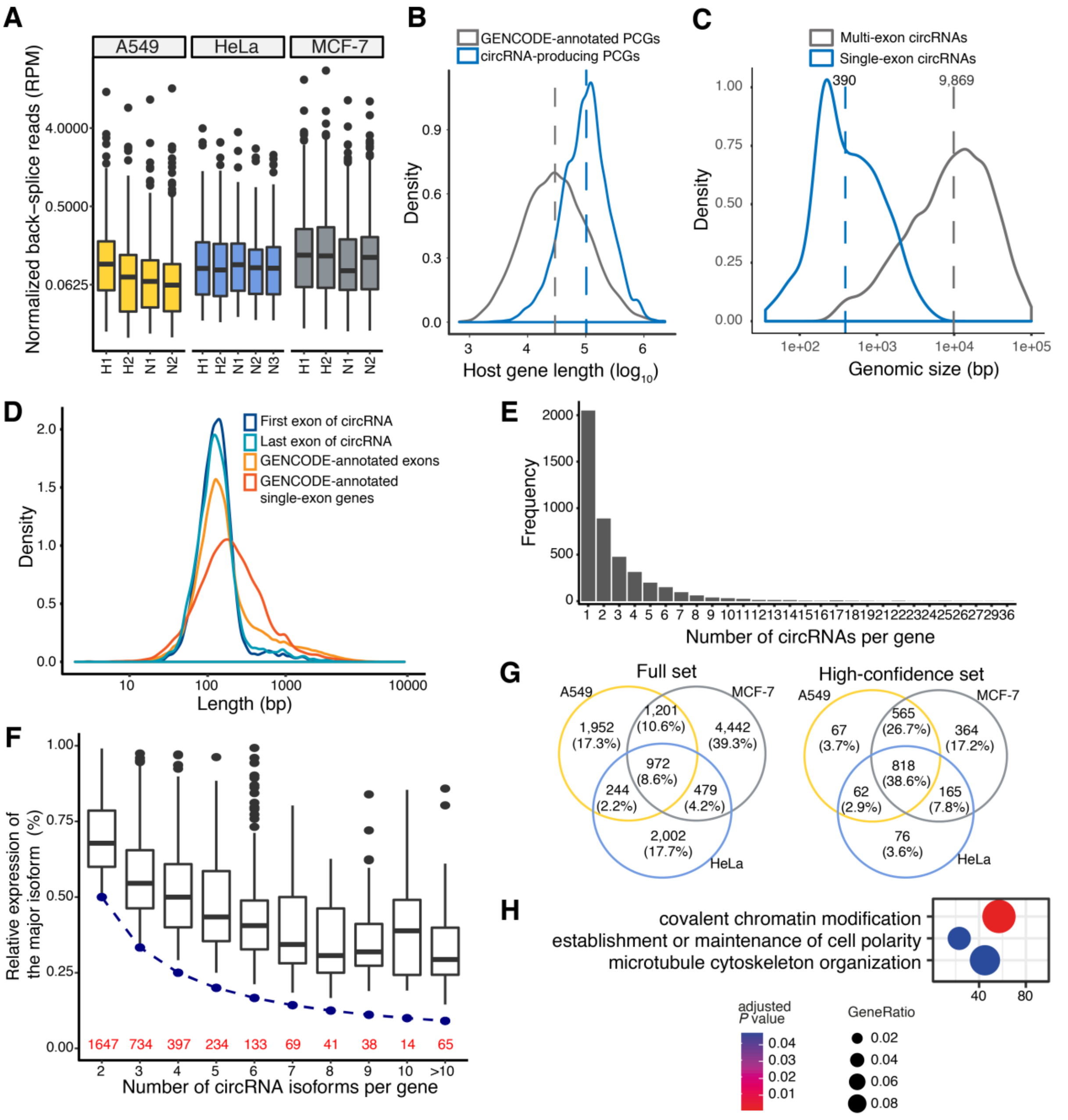

Supplementary Figure 3. Characterization of the circRNA catalogue. (**A**) circRNAs in MCF-7 generally show higher back-splice read counts. Boxplot shows the distribution of normalized back-splice read counts (reads per million, RPM) in each replicate for the three cell lines. (**B**) circRNA-producing genes are significantly longer than average protein-coding genes (*P* value < 2.2e-16, Wilcoxon rank sum test). Density plot compares the length distribution of protein-coding genes with circRNAs compared to all annotated protein-coding genes (GENCODE v24). Dashed lines indicate medians. (**C**) Density plot depicts the distribution of genomic sizes (i.e. genomic distance between back-splice sites) of multi-exon and single-exon circRNAs. Median values are given above dashed lines. (**D**) Back-splicing exons are not longer than average exons. Density plot compares the length distribution of circularized exons (i.e. first and last exons of circRNAs) with all annotated internal exons (GENCODE v24), distinguishing between exons from multi-exon and single-exon genes. (**E**) A substantial number of host genes produce more than one circRNA isoform. Histogram

summarizes number of circRNAs per host gene. We note that the frequency of alternative back-splicing is likely underestimated, as our analysis is solely based on back-splice junctions and does not consider possible internal alternative splicing events. (**F**) Most host genes produce few predominant circRNA isoforms. Boxplot compares the relative abundance of circRNA isoforms produced from a given gene to the expected frequency based on equal proportions. Genes were stratified by the number of associated circRNA isoforms, grouping genes with more than ten circRNA isoforms. Blue line and dots represent the expected relative abundance computed from total circRNA isoforms from a given gene (1/number of circRNA isoforms). (**G**) circRNAs detected in A549, HeLa and MCF-7 cells with an additional filter on host gene expression (TPM ≥ 5 in any sample of a given cell line). Venn diagrams depict the overlap between the three cell lines for the full catalogue of predicted circRNAs (n = 12,006) (left), or the high-confidence set of circRNAs (at least five back-splice reads in any two samples; n = 2,205) (right). (**H**) Enrichment of GO terms (Biological Process) for 690 circRNA host genes. Visualization as in Supplementary Figure 1C.

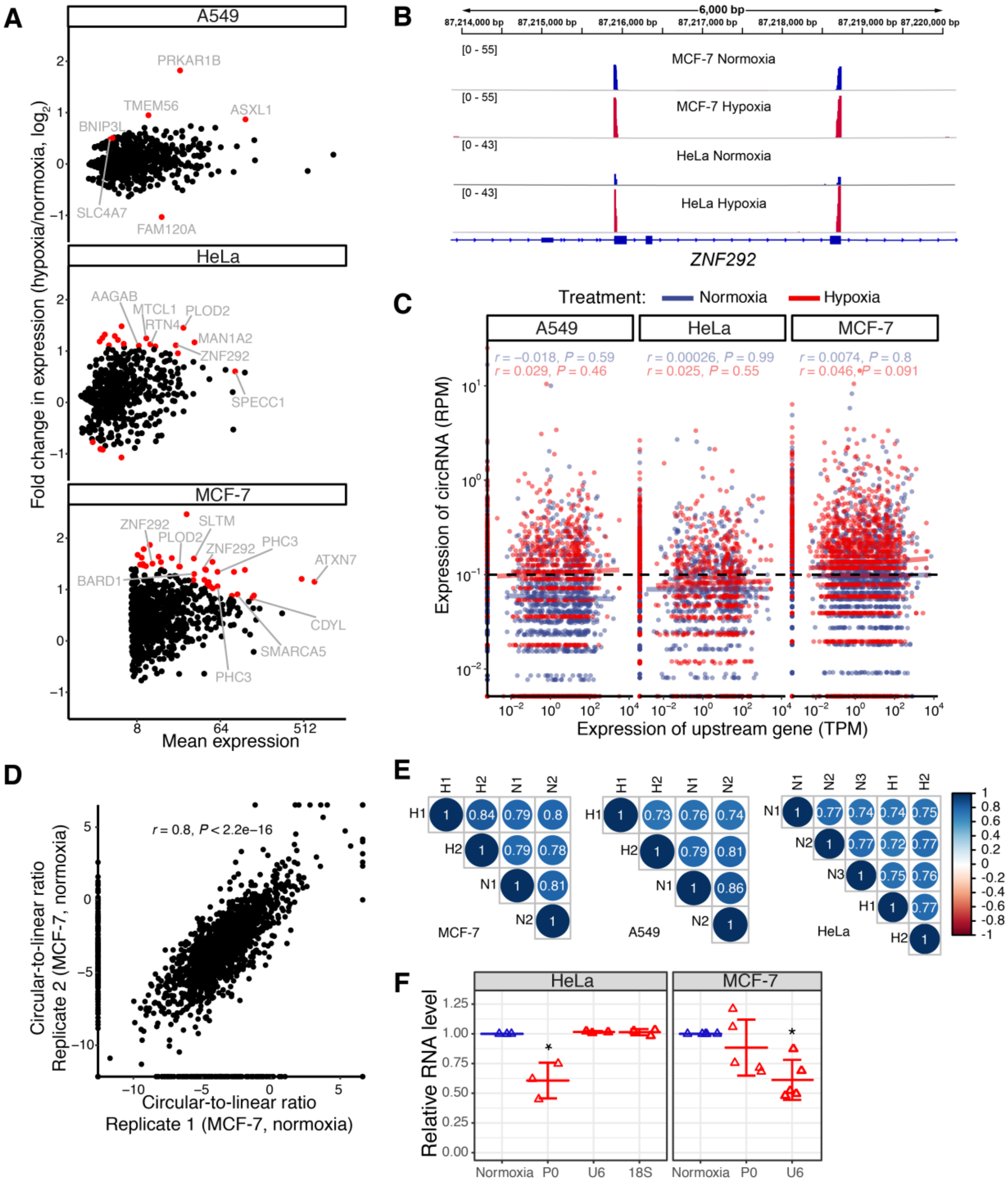

Supplementary Figure 4. Expression of circRNAs between replicates and in relation to the upstream gene. (**A**) MA plots show the average expression of circRNAs in the three cell lines against log2-transformed moderated fold changes in expression (hypoxia over normoxia, taken from DESeq2). 64 circRNAs significantly change in abundance upon hypoxia (FDR < 0.05) and are highlighted in red. Only high-confidence circRNAs with at least five reads in any two samples of a single cell line were tested for differential expression. (**B**) circZNF292 (exonic) is upregulated under hypoxia in MCF-7 and HeLa cells. Genome browser view of exons 2-5 of the gene *ZNF292*. Chimeric alignments (back-splice reads) from RNA-Seq data for MCF-7 and HeLa cells under normoxic and hypoxic conditions are shown, similarly to Figure 4B. (**C**) circRNA expression is not influenced by read-through

from the upstream gene. Scatter plot compares the expression of circRNAs (in back-splice reads per million, RPM) to the expression of the gene encoded upstream of the circRNA host gene in the genome (in transcripts per million, TPM). Mean expression across replicates is shown for each cell line under hypoxic (blue) and normoxic (red) conditions. Linear regression lines and Pearson correlation coefficients with associated *P* values are shown. (**D**) Back-splicing rates are consistent between replicates. Scatter plot compares circular-to-linear ratios (CLR) of all high-confidence circRNAs between two replicate samples with MCF-7 cells under normoxic conditions. Pearson correlation coefficient and associated *P* value are given above. (**E**) Matrix of pairwise Pearson correlation coefficients (color-coded) between all samples for each cell line. **(F)** Quantitative PCR (RT-qPCR) measurements of three housekeeping genes in HeLa and MCF-7 cells. *P0* levels remain stable in MCF-7 cells and are significantly changed upon hypoxia in HeLa cells. Both U6 snRNA and 18S rRNA levels remain constant upon hypoxia in HeLa cells (n = 3-5, * $P < 0.05$).

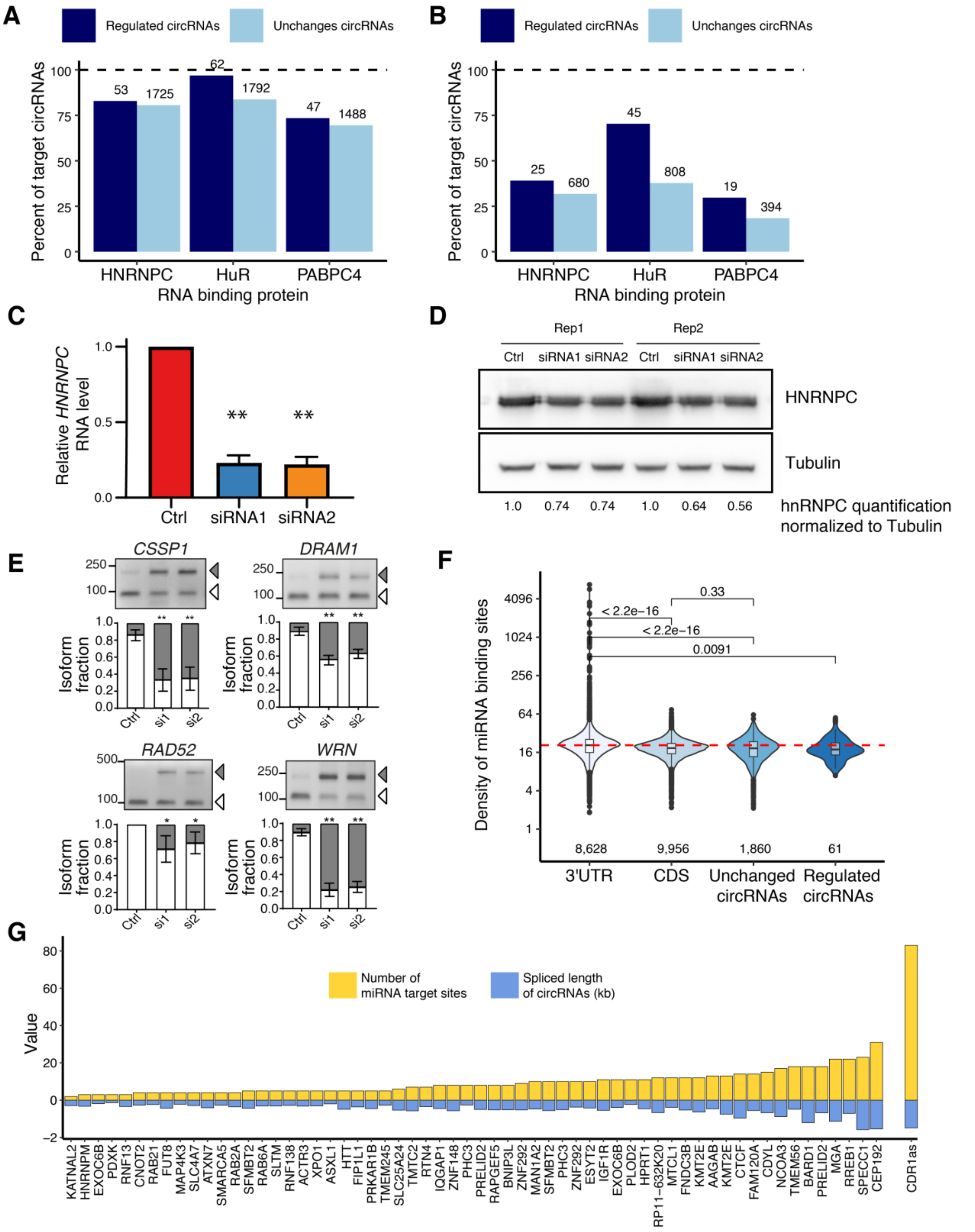

Supplementary Figure 5. Characterization of RBPs and miRNAs related to circRNA biogenesis and function. (**A,B**) *In silico* predictions indicate individual RBP binding sites (**A**) and flanking binding site pairs (**B**) at a large fraction of the 64 hypoxia-regulated and remaining unchanged circRNAs (high-confidence set) for HNRNPC, HuR and PABPC4. Barchart shows number of circRNAs with predicted binding sites of a given RBP. Dashed lines mark 100%. Number of circRNAs in each category given above each bar. Predictions

for HNRNPCL1 were removed since the motif is almost identical to HNRNPC. (**C**) RT-qPCR shows efficient depletion of *HNRNPC* RNA levels in HeLa cells upon knockdown with two independent siRNAs (siRNA1 and siRNA2). *HNRNPC* levels were normalized to U6 snRNA and related to control levels (Ctrl) with unspecific siRNA (n = 3, ** $P < 0.01$, Student's t-test). (**D**) HNRNPC is modestly downregulated at the protein level. Western blot quantifies the amount of HNRNPC protein in the same samples as in (C). (**E**) Control RT-PCR experiments analyzing four splicing changes of exons that are known to be regulated by HNRNPC (Zarnack et al., 2013). Electrophoresis gels of the PCR products (top) are shown for each validated exon. The sizes of the quantified fragments are indicated on the left of the gel image. The inclusion and exclusion isoforms are marked on the right in grey and white, respectively. For each exon, the signal quantification is shown in the barchart below the gel (n = 3, * $P < 0.05$, ** $P < 0.01$, Student's t-test). Data are shown as mean ± SD. (**F**) circRNAs are not enriched for miRNA target sites. Violin plot shows distribution of predicted miRNA target sites per region for circRNAs from the high-confidence set with assigned parental transcript, as well as 10,000 randomly selected coding sequences (CDS) and 3'UTR sequences (GENCODE v24). *P* values are shown for selected comparisons (two-sample Wilcoxon test). (**G**) None of the hypoxia-regulated circRNAs harbors an excess of miRNA target sites. Barchart contrasts the number of predicted miRNA target sites to the predicted circRNA length. CDR1as/ciRS-7 is shown for comparison.
